# Interaction of the Warsaw breakage syndrome DNA helicase DDX11 with the replication fork-protection factor Timeless promotes sister chromatid cohesion

**DOI:** 10.1371/journal.pgen.1007622

**Published:** 2018-10-10

**Authors:** Giuseppe Cortone, Ge Zheng, Pasquale Pensieri, Viviana Chiappetta, Rosarita Tatè, Eva Malacaria, Pietro Pichierri, Hongtao Yu, Francesca M. Pisani

**Affiliations:** 1 Istituto di Biochimica delle Proteine, Consiglio Nazionale Ricerche, Naples, Italy; 2 Howard Hughes Medical Institute, Department of Pharmacology, University of Texas Southwestern Medical Center, Dallas, TX, United States of America; 3 Istituto di Genetica e Biofisica "Adriano Buzzati Traverso", Consiglio Nazionale Ricerche, Naples, Italy; 4 Istituto Superiore di Sanità, Dipartimento Ambiente e Salute, Rome, Italy; Columbia University, UNITED STATES

## Abstract

Establishment of sister chromatid cohesion is coupled to DNA replication, but the underlying molecular mechanisms are incompletely understood. DDX11 (also named ChlR1) is a super-family 2 Fe-S cluster-containing DNA helicase implicated in Warsaw breakage syndrome (WABS). Herein, we examined the role of DDX11 in cohesion establishment in human cells. We demonstrated that DDX11 interacts with Timeless, a component of the replication fork-protection complex, through a conserved peptide motif. The DDX11-Timeless interaction is critical for sister chromatid cohesion in interphase and mitosis. Immunofluorescence studies further revealed that cohesin association with chromatin requires DDX11. Finally, we demonstrated that DDX11 localises at nascent DNA by SIRF analysis. Moreover, we found that DDX11 promotes cohesin binding to the DNA replication forks in concert with Timeless and that recombinant purified cohesin interacts with DDX11 *in vitro*. Collectively, our results establish a critical role for the DDX11-Timeless interaction in coordinating DNA replication with sister chromatid cohesion, and have important implications for understanding the molecular basis of WABS.

## Author summary

Chromosomes are DNA molecules that contain the genetic information. During replication, the two sister DNA molecules covered by proteins (sister chromatids) are held together by many copies of a ring-like protein complex named cohesin, in a process called sister-chromatid cohesion. Before a cell divides, the cohesin rings are removed from the two sister chromatids to allow their migration towards the opposite poles of the dividing mother cell. At the end of this process, the two daughter cells have inherited a complete set of chromosomes. Before the next cell division, chromosomes are duplicated with high speed and fidelity. This important task is performed by the DNA replication machinery, a sophisticated apparatus made of several enzymes and proteins. In the present study, we have demonstrated that DDX11 and Timeless, two subunits of the DNA replication machinery, recruit the cohesin rings to promote their stable binding to the newly duplicated chromosomes, that is the establishment of sister-chromatid cohesion. In human cells that were genetically engineered to reduce the level of DDX11, we observed that sister-chromatid cohesion was loosened and association of cohesin to chromosomes was reduced. Our experimental results contribute to our understanding of the molecular mechanisms underlying the functional coupling between DNA replication and sister-chromatid cohesion in human cells.

## Introduction

Cohesion is the process that ensures tethering of newly replicated sister chromatids until they separate in metaphase [1]. This process is mediated by cohesin, an evolutionarily conserved hetero-tetrameric complex (made of Smc1, Smc3, Scc1 and either SA1 or SA2 subunits), which has a ring-like structure and is believed to encircle DNA [2–4]. Several proteins interact with cohesin during different phases of the cell cycle and regulate its association with chromatin.

In mammalian cells, cohesin is loaded onto DNA in telophase by the action of the loader complex (Scc2-Scc4) [5]. During G1 phase, cohesin association with chromatin is dynamic and cohesin can be unloaded by the activity of the Wapl-Pds5 complex. In S phase (and subsequent G2) binding of cohesin to chromatin becomes stable, as a consequence of acetylation of the Smc3 subunit by two dedicated acetyltransferases (Esco1 and Esco2) [6–7]. This process, known as cohesion establishment, renders cohesin resistant to the action of Wapl-Pds5 and is believed to take place at the replication fork [8–10].

Genetic inactivation of a number of DNA replication factors results in cohesion defects in yeast and in mammalian cells [11–12]. An important role in the chromosomal cohesion process is played by Timeless (Tof1/Swi1 in yeast), which, together with Tipin (Csm3/Swi3 in yeast) and Claspin (Mrc1 in yeast), forms the replication *f*ork-*p*rotection *c*omplex (FPC). The FPC has multiple important functions for maintaining genome stability during DNA replication [13]. First, the FPC is a mediator of the S phase checkpoint promoting ATR-mediated Chk1 phosphorylation. Second, the FPC plays roles that are independent of the S phase checkpoint. It associates with the advancing replisomes and prevents uncoupling of replicative DNA polymerases from the DNA helicase, when DNA synthesis is halted at sites of DNA damage or at natural replication fork barriers. Lastly, FPC components promote chromosomal cohesion in various systems, including yeasts [14–15], *Caenorhabditis elegans* [16], *Xenopus laevis* egg extracts [17–18] and human cells [19–20].

Genetic studies in yeast have revealed a functional link between the FPC and the cohesion establishment factor Chl1 (*Ch*romosome *l*oss 1 protein) [14, 21–22]. Chl1, also known as ChlR1 or DDX11 in metazoans, is a *s*uper-*f*amily 2 (SF2) ATP-dependent DEAH-box DNA helicase that unwinds DNA with a 5’-to3’ directionality [23]. Human DDX11 shares sequence similarity with the Fe-S cluster-containing DNA helicases FANCJ, XPD and RTEL1. All of these helicases play important roles in genome stability maintenance and are implicated in rare genetic syndromes and cancer development [24–25]. DDX11 is genetically linked to the *Wa*rsaw *b*reakage *s*yndrome (WABS), a rare hereditary disease. WABS-affected individuals display a complex pattern of clinical manifestations, including reduced growth, skin rash, heart defects, deafness, and intellectual disability. At the cytological level, WABS patient cells exhibit increased drug-induced chromosomal breakage and sister chromatid cohesion defects [26–27]. We have previously demonstrated that DDX11 and Timeless physically and functionally interact and operate in concert to preserve replication fork progression in stressful conditions in HeLa cells [28]. Nonetheless, the precise molecular mechanism, by which DDX11 and Timeless cooperate with other components of the replication machinery and/or the cohesin complex to promote genomic stability and sister chromatid cohesion, has not yet been elucidated.

Herein, we identify a Timeless-binding motif in DDX11 and show that mutations of this sequence compromise the DDX11-Timeless interaction. We demonstrate that DDX11 mutants defective in Timeless binding are unable to rescue sister chromatid cohesion defects of DDX11-depleted HeLa cells. Conversely, DDX11 helicase-dead mutants partially revert the loss-of-cohesion phenotype of these cells. These results suggest that the interaction of DDX11 with Timeless is critical for sister chromatid cohesion. Besides, we demonstrate that DDX11 and cohesin associate with replication forks in HeLa cells and this association is reduced when DDX11 is down-regulated. In addition, we show that DDX11 interacts with the cohesin complex in cell extracts and *in vitro*. Overall, our data suggest that DDX11 has a scaffolding function in sister chromatid cohesion by anchoring the cohesin complex to the replication machinery and underscore the importance of the DDX11-Timeless interaction for linking replication fork progression to chromosomal cohesion in human cells.

## Results

### DDX11 binds Timeless through a conserved sequence motif

We previously demonstrated that human DDX11 and Timeless directly interact and collaborate to preserve replication fork stability [28]. To identify DDX11 residues responsible for Timeless binding, we carried out an analysis based on tiling peptide microarrays that covered the entire length of the DDX11 sequence. These arrays consisted of 454 15-residue long peptides that were "printed" in duplicate on a glass slide. They were probed with purified recombinant Flag-Timeless and subsequently detected with a fluorescently labelled anti-Flag antibody. As the negative control, an identical peptide array was subjected to mock incubation with the anti-Flag antibody, but without Flag-Timeless.

**Fig 1:**
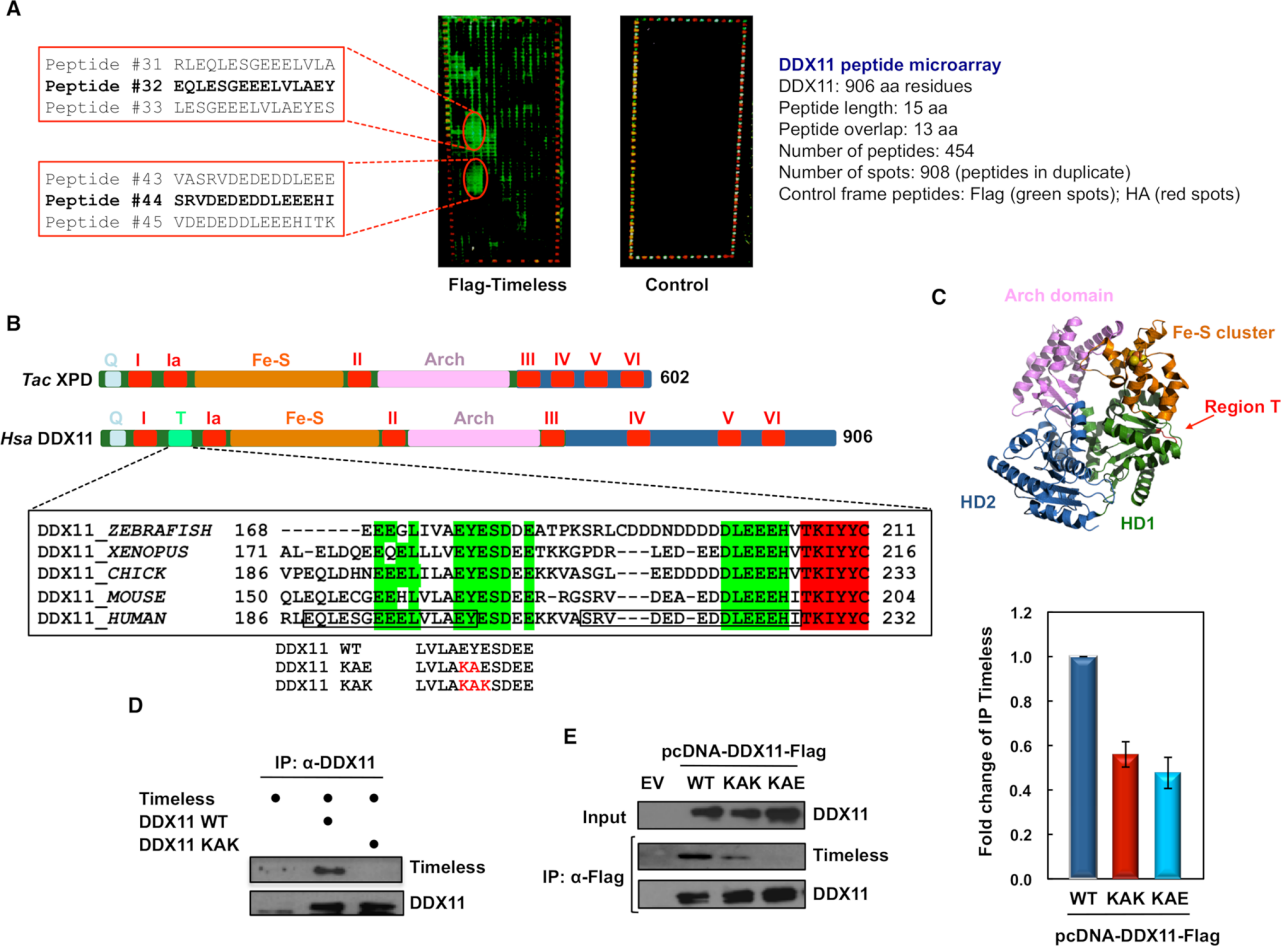
Identification of DDX11 Timeless-binding sites. ***A***) Overlapping peptide micro-arrays that cover the full-length DDX11 sequence (amino acid residues 1–906) were probed with (on *left*) or without (control micro-array on *right*) recombinant purified Flag-tagged Timeless and detected with a mixture of Cy3-labelled anti-Flag antibody and Cy5-labelled anti-HA antibody. Images of the probed arrays obtained with a high-resolution fluorescence scanner are shown. The sequence of DDX11 peptides interacting with Timeless are reported on *left*. Peptides # 32 and # 44 showing the strongest binding signal in each interaction spot are highlighted in *bold*. ***B***) Schematic representation of the polypeptide chain of *Homo sapiens* (*Hsa*) DDX11 and *Thermoplasma acidophilum* (*Tac*) XPD, both belonging to the group of SF2 DNA helicases with a Fe-S cluster. Conserved helicase motifs (from I to VI) are indicated in *red*. Other sequence motifs are indicated with different colours. Abbreviations used are: *Q*, for Q motif; *Fe-S*, for Fe-S cluster; *Arch*, for Arch domain. A multiple sequence alignment of DDX11 Region T from various vertebrates is reported. Highly conserved residues are in *bold*; invariant residues are highlighted in *green*. Adjacent DNA helicase motif Ia is highlighted in *red*. The sequences of human DDX11 peptides # 32 and # 44 are boxed. Region T mutations to generate the DDX11 KAE and KAK mutants are indicated in *red*. ***C***) The *Tac* XPD DNA helicase crystal structure (PDB code: 4a15_A, [29]) is shown. RecA-*h*omology *d*omain 1 and 2 (*HD1* and *HD2*) are in *green* and *blue*, respectively. Fe-S cluster and Arch domain are depicted in *orange* and *pink*, respectively. Iron and sulphur atoms are shown and coloured in *orange* and *yellow*, respectively. Putative position of Region T containing the Timeless-binding sites is shown in *red*. ***D***) Co-pull down analyses on mixtures of the indicated purified recombinant proteins were carried using an anti-DDX11 antibody bound to Protein A Sepharose beads. Pulled down samples were subjected to immuno-blot analysis to detect the indicated proteins. ***E***) HEK 293T cells were transiently transfected with an empty vector (*EV*) or a vector expressing Flag-tagged wild type (*WT*) DDX11 and its mutants (*KAK* and *KAE*). 48 hr post-transfection, whole cell extracts were subjected to immuno-precipitation with anti-Flag M2 agarose beads. Western blot analyses of the pulled down samples were carried out and endogenous Timeless was detected using specific antibodies. Immuno-blot experiments were carried out on properly diluted samples and the ImageJ software was used for quantitative analyses of protein bands. Level of immuno-precipitated Timeless was normalized to pulled down Flag-tagged DDX11 in each sample. Means with standard errors of three independent experiments are shown. According to Student’s *t*-test, a value of *P* < 0.005 was calculated for the following dataset pairs: Flag-tagged DDX11 WT versus KAK and KAE.

As shown in Fig 1A, two main interaction spots were identified, which were not present in the negative control. These spots were centred around Peptide # 32 and # 44, which map to the N-terminal portion of DDX11 between helicase motifs I and Ia (Fig 1B). A multiple sequence alignment revealed that this region of human DDX11 (here named Region T, residues 65–225) forms an insertion that is shared only by FANCJ, but not other SF2 Fe-S DNA helicases (see S1 Fig). According to a DDX11 three-dimensional model based on the *Thermoplasma acidophilum* XPD crystal structure [29], Region T is predicted to reside on the protein surface in the RecA-*h*omology *d*omain 1 (HD1; see Fig 1C).

To identify amino acid residues critical for Timeless binding, we used microarrays containing a full substitution scan of DDX11 Peptide # 32. In these arrays, each residue of Peptide # 32 was substituted with all 20 natural amino acids. We found that substitution of the two C-terminal residues of Peptide # 32 (corresponding to Glu^201^ and Tyr^202^ of full-length DDX11) with lysine completely abolished the interaction with Timeless (S2 Fig). Other changes of the same residues had a less drastic effect on Timeless binding.

Then, we carried out site-directed mutagenesis studies of full-length DDX11 to validate the importance of the above residues for Timeless binding (Fig 1D and 1E). We noticed that DDX11 Glu^201^ and Tyr^202^ belong to a short highly conserved sequence that we named "EYE" motif. A multiple sequence alignment revealed that this motif is invariant in DDX11 orthologs from vertebrates, whereas it is only partially conserved in DDX11 proteins from fruit fly, worm, budding yeast and fission yeast (S3B Fig). Residues of human DDX11 "EYE" motif were substituted to produce the mutants that were named DDX11 KAE and KAK. We observed an almost complete loss of interaction between Timeless and the DDX11 KAK mutant, when co-pull down experiments were performed *in vitro* on mixtures of these proteins produced in the recombinant form (Fig 1D). Moreover, interaction of the DDX11 KAE and KAK mutants with the endogenous Timeless was examined by co-immuno-precipitation experiments performed on whole extracts of HEK 293T cells ectopically expressing these DDX11 mutant forms. These analyses revealed that the above DDX11 amino acid changes strongly reduced Timeless binding in human cells (Fig 1E). Therefore, the conserved "EYE" motif of DDX11 is critical for Timeless binding, although we cannot completely exclude that other contact sites could exist between the two proteins. Besides, as the association between the DDX11 KAK and KAE mutants and Timeless is not completely abolished in whole cell extracts, additional protein factors could mediate DDX11:Timeless interaction *in vivo*.

### The DDX11-Timeless interaction promotes sister chromatid cohesion

**Fig 2:**
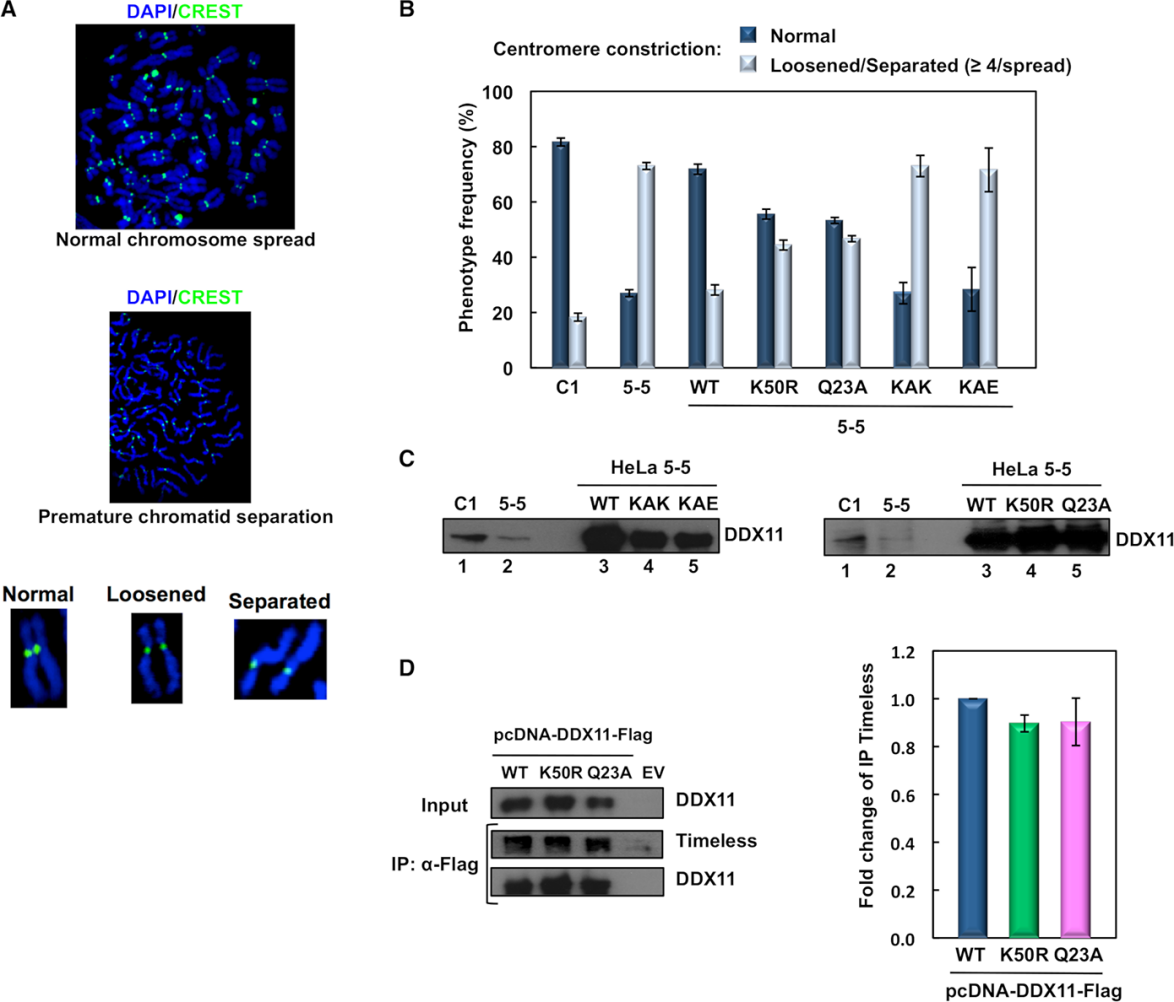
DDX11:Timeless interaction is critical for centromeric chromosomal cohesion in M phase cells. ***A***) Representative images of HeLa metaphase chromosome spreads. Typical chromosome morphologies are shown after magnification. Spreads were stained with DAPI (*blue*) and the human centromere/kinetochore marker CREST (*green*). ***B***) Plot showing frequency of the indicated chromosome configurations in spreads prepared from control (*C1*) and DDX11-depleted (*5–5*) HeLa cells that were transfected with pcDNA 3.0 vector derivatives expressing DDX11 wild type (*WT*) and its indicated mutants. Spreads with at least four chromosomes with loosened/separated chromatids were arbitrarily classified as defective in cohesion. At least 50 spreads were examined for each condition in two independent experiments. Error bars represent standard deviation. According to Student’s *t*-test, values of *P* < 0.0005 were calculated for the following dataset pairs: 5-5/WT versus 5-5/K50R, 5-5/Q23A, 5-5/KAK, 5-5/KAE. ***C***) Evaluation of DDX11 expression level by Western blot analysis of extracts from the indicated HeLa cell lines, which were collected after 24-h post-transfection, 16-h thymidine block and 9-h release in fresh medium, as described in the *Materials and Methods* section. Samples containing 20 μg (*lanes 1* and *2*) or 5 μg (*lanes 3*, *4* and *5*) of total protein from whole cell extracts were employed for the immuno-blot analyses. Quantification of band intensity by ImageJ indicated a relative ratio of 3:1 and 4:1 for DDX11 in C1 versus 5–5 cells (immuno-blot on *left* and *right*, respectively); 1:0.67:0.60 for DDX11 in 5–5 HeLa cells transfected with vector expressing wild type versus KAK versus KAE protein; 1:1:1 for DDX11 in HeLa 5–5 cells transfected with vector expressing wild type versus K50R versus Q23A protein. ***D***) DDX11 helicase-dead mutants binds Timeless in cell extracts. HEK 293T cells were transiently transfected with an empty vector (*EV*) or a vector expressing Flag-tagged wild type (*WT*) DDX11 and its helicase-dead mutants (*K50R* and *Q23A*). 48 hr post-transfection, whole cell extracts were subjected to immuno-precipitation with ant-Flag M2 agarose beads. Western blot analyses were carried to detect endogenous Timeless protein in the pulled down samples using specific antibodies. Immuno-precipitation experiments were done at least in triplicate and quantitative analyses were carried out on properly diluted pulled down samples using the ImageJ software. Level of immuno-precipitated Timeless was normalized to pulled down Flag-tagged DDX11 in each sample. Means with standard errors of three independent experiments are shown. According to Student’s *t*-test, not significant *P* values were calculated for the following dataset pairs: Flag-tagged DDX11 WT versus K50R (*P* < 0.025) and Q23A (*P* < 0.25).

We then examined the relevance of the DDX11-Timeless interaction in sister chromatid cohesion in interphase and mitosis. These analyses were carried out in a HeLa cell line where DDX11 was stably knocked-down. This cell line (named HeLa 5–5) was established by infection with a pantropic retrovirus (pSuper-Retro-Puro) that expresses a shRNA targeting the DDX11 coding sequence. At the same time, a control cell line (named HeLa C1) was obtained by infection with an empty retrovirus, as previously described [30–31]. Centromeric cohesion was examined in metaphase chromosome spreads by indirect immuno-fluorescence with the human CREST antibody that specifically recognizes inner centromere/kinetochore proteins (Fig 2A and 2B). As expected, the majority (about 82%) of control cells displayed sister chromatid pairs with a typical tight primary constriction. By contrast, a high proportion (about 73%) of DDX11-depleted HeLa cells had metaphase chromosomes with a loosened centromere constriction. A small fraction of cells gave rise to metaphase chromosome spreads with a total premature chromatid separation. These findings are consistent with previous reports showing that DDX11 is required for proper chromosomal cohesion [30, 32].

The DDX11-depleted HeLa cells were transiently transfected with vectors expressing wild type DDX11 and the KAE and KAK mutants to assess the ability of these proteins to rescue the observed chromosomal cohesion defect (Fig 2B). As shown in Fig 2C, Flag-tagged DDX11 mutants were expressed at a level that was comparable with that of the wild type protein. The complementation assays indicated that ectopically expressed wild type DDX11 was able to efficiently rescue the loss-of-cohesion phenotype of the DDX11-depleted cells, with about 72% of spreads having cohered chromatid pairs. In contrast, over-expressed DDX11 KAE and KAK mutants did not revert the chromosomal cohesion defect and about 71–73% of the examined spreads displayed chromosomal cohesion anomalies. These results indicate that the interaction of DDX11 with Timeless is needed for proper sister chromatid cohesion.

Previous biochemical studies revealed that substitution of Lys^50^ with Arg in the helicase motif I (Walker A) of human DDX11 (DDX11 K50R) abolished ATP binding/hydrolysis and DNA unwinding, but not DNA-binding activity [33]. Besides, human DDX11 with substitution of Gln^23^ with Ala in the so-called conserved Q motif (DDX11 Q23A) was reported to be completely unable to bind/hydrolyze ATP and bind/unwind DNA [34]. We analyzed the ability of these two DDX11 helicase-dead mutants to correct the chromosomal cohesion defects observed in the DDX11-depleted HeLa cells. Our complementation studies revealed that either DDX11 K50R or Q23A was able to correct the centromeric cohesion defect of DDX11-depleted HeLa cells, although not as efficiently as the wild type protein: about 55% and 53% of chromosome spreads showed normal chromosomal pairing, respectively (Fig 2B). We then examined the Timeless-binding capability of DDX11 K50R and Q23A helicase-dead mutants by co-pull down experiments in whole cell extracts, and found that it was not reduced as compared to wild type DDX11 (Fig 2D). These results revealed that DDX11 has a role in sister chromatid cohesion that is not strictly dependent on its catalytic functions (ATP-binding/hydrolysis and DNA-binding/unwinding).

We also analysed the effect of Wapl down-regulation in DDX11-depleted HeLa cells and found that the percentage of mitotic cells with premature chromatid separation was reverted to a normal level in cells where expression of the cohesin releasing factor was knocked-down (see S4A and S4B Fig). Thus, these results suggest that at least a function of DDX11 in cohesion establishment is to counteract Wapl. We note, however, that the depletion of DDX11 by siRNA is incomplete. It remains to be tested whether Wapl depletion can rescue the cohesion defects caused by the complete loss of DDX11. Besides, we found that down-regulation of DDX11 did not substantially affect the level of acetylated Smc3 in HeLa cells (see S4C Fig).

**Fig 3:**
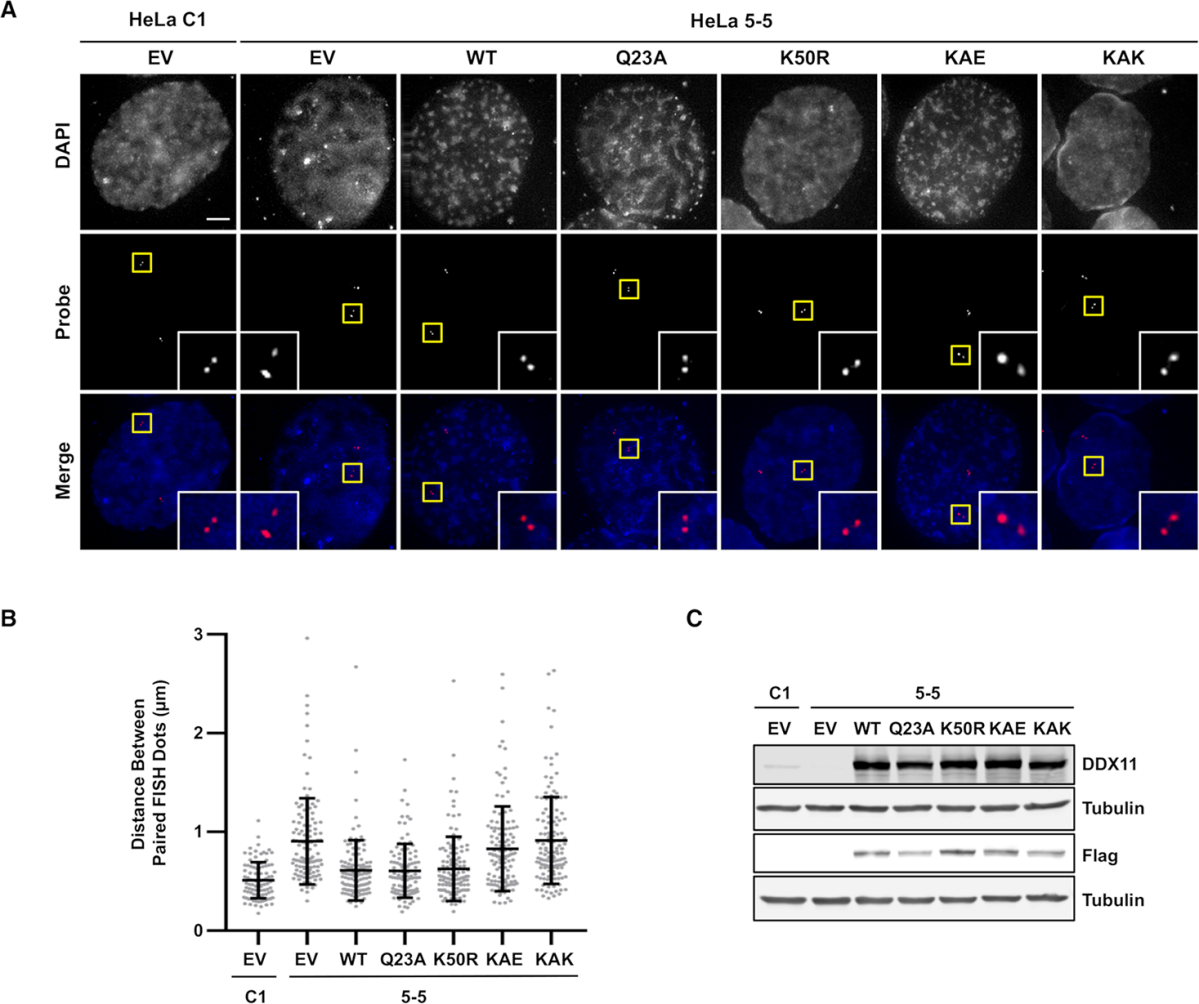
Complementation assays of cohesion defects by FISH analysis in G2 cells. ***A*)** Representative images of G2-enriched control (*HeLa C1*) and DDX11-depleted (*HeLa 5–5*) HeLa cells, which were transfected with empty vector (*EV*) or constructs expressing DDX11 wild type and its indicated mutants, and stained with DAPI (*blue* in merge) and the FISH probe (*red* in merge). Cells were treated with thymidine for 16–18 h and released into fresh medium for 4 h before fixation. Selected paired FISH signals are magnified in *inset*. *Scale bar*, 5 μm. ***B***) Quantification of the distances between paired FISH signals in G2-enriched HeLa cells transfected with plasmids expressing the indicated DDX11 proteins. Each dot in the graph represents the distance between one pair of FISH signals in cell. Mean values with standard deviations are indicated (C1, n = 95; 5–5, n = 122; WT, n = 123; Q23A, n = 106; K50R, n = 126; KAE, n = 125; KAK, n = 137). According to Student’s *t*-test, a value of *P* < 0.0001 was calculated for the following dataset pairs: C1/EV versus 5-5/EV, 5-5/KAE, 5-5/KAK; 5-5/EV versus 5-5/WT, 5-5/Q23A, 5-5/K50R; 5-5/WT versus 5-5/KAE, 5-5/KAK; 5-5/Q23A versus 5-5/KAE, 5-5/KAK; 5-5/K50R versus 5-5/KAE, 5-5/KAK; a value of *P* < 0.0049 for C1/EV versus 5-5/WT; a value of *P* < 0.0047 for 5-5/Q23A; a value of *P* < 0.0022 C1/EV versus 5-5/K50R. Not significant *P* values were calculated for the following dataset pairs: 5-5/EV versus 5-5/KAE (*P* = 0.1727), 5-5/KAK (*P* = 0.8682); 5-5/WT versus 5-5/Q23A (*P* = 0.8818), 5-5/K50R (*P* = 0.7112); 5-5/Q23A versus 5-5/K50R (*P* = 0.6065); 5-5/KAE versus 5-5/KAK (*P* = 0.1174). ***C***) Evaluation of DDX11 expression level by Western blot analysis of extracts from the indicated HeLa cells treated as above described.

Next, we evaluated the ability of the above DDX11 mutants to revert the chromosomal cohesion defects of DDX11-depleted cells in G2 phase by a *f*luorescence *i**n* *s**itu* *h*ybridization (FISH) assay, using a probe specific for a chromosome 3 locus (Fig 3A–3C). After an overnight thymidine block, HeLa cells were released in fresh medium for 4 hr to enrich them in late S/G2 phase. Distance between FISH signals in G2 phase nuclei was measured to examine the cohesion status in single cells. We found that cohesion defects caused by DDX11 loss were rescued by the wild type protein and the helicase-dead mutants (K50R and Q23A). In contrast, the Timeless-binding defective mutants (KAE and KAK) were unable to restore a normal distance between paired FISH dots (Fig 3B). These results revealed that the DDX11-Timeless interaction is critical for chromosomal cohesion even in interphase nuclei.

### DDX11 is required for cohesin loading onto chromatin during S phase

**Fig 4:**
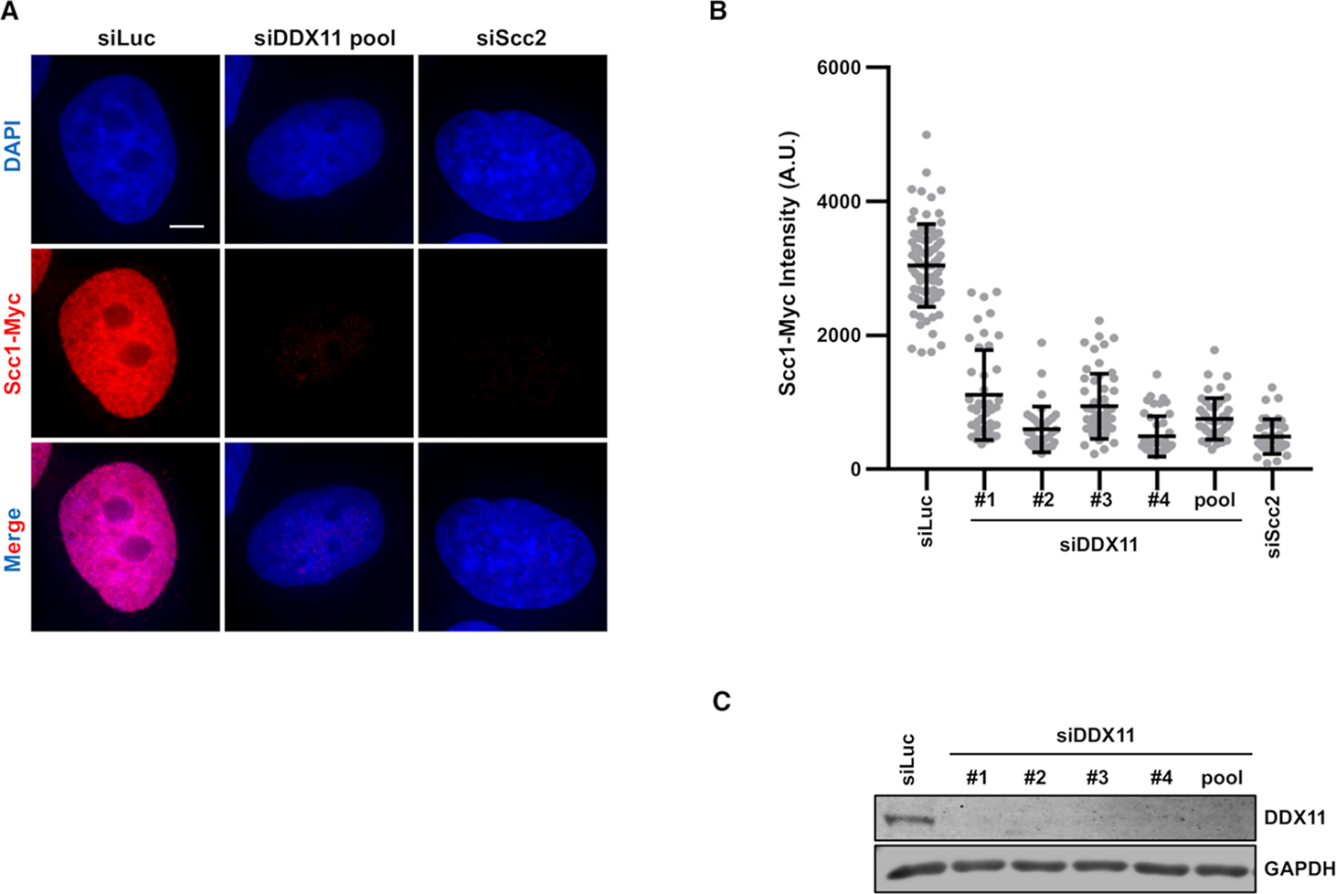
DDX11 promotes cohesin loading onto chromatin in S phase HeLa cells. ***A***) DAPI (*blue*) and anti-Myc (*red*) staining of HeLa cells that stably expressed Scc1-Myc. Cells were transfected with the indicated siRNAs and arrested in early S phase with thymidine. Scale bar, 5 μm. ***B***) Quantification of the Scc1-Myc chromatin intensities of cells in ***A***. Each dot in the graph represents a single cell. Mean values with standard deviations are reported (siLuc, n = 82; siDDX11: #1, n = 44; #2, n = 35; #3, n = 53; #4, n = 45; pool, n = 46; siScc2, n = 39). According to Student’s *t*-test, a value of *P* < 0.0001 for the following dataset pairs: HeLa/siLuc versus HeLa/siDDX11 # 1, # 2, # 3, # 4, pool, HeLa/siScc2 ***C***) Extracts of HeLa cells transfected with the indicated siRNAs were analyzed by immunoblot with the indicated antibodies.

We have recently shown that Mcm2–7-dependent cohesin loading in early S phase is critical for cohesion establishment in human cells [35]. We checked if DDX11 is also required for cohesin loading in S phase. In HeLa cells stably expressing Scc1-Myc we depleted DDX11 using siRNA and examined the level of chromatin-bound Scc1-Myc. In cells arrested in early S phase with thymidine treatment, the intensity of Scc1-Myc was consistently reduced with four individual DDX11 siRNAs and of all four siRNAs (Fig 4A and 4B). Depletion of DDX11 with each DDX11 siRNA was efficient (Fig 4C). Then, we extended this analysis to the cohesin SA2 subunit and found that its association to chromatin was also reduced in DDX11-depleted HeLa cells synchronised in S phase (S5 Fig). Moreover, rescue experiments indicated that Scc1-Myc loading onto chromatin was partially restored by the DDX11 helicase-dead mutants (K50R and Q23A), but not by the Timeless-binding defective mutants (KAE and KAK) (S6 Fig).

Collectively, these results suggest that DDX11 promotes stable association of cohesin to chromatin during S phase in a way that is dependent on its direct interaction with Timeless.

### DDX11 physically interacts with cohesin and localises at replication forks

**Fig 5:**
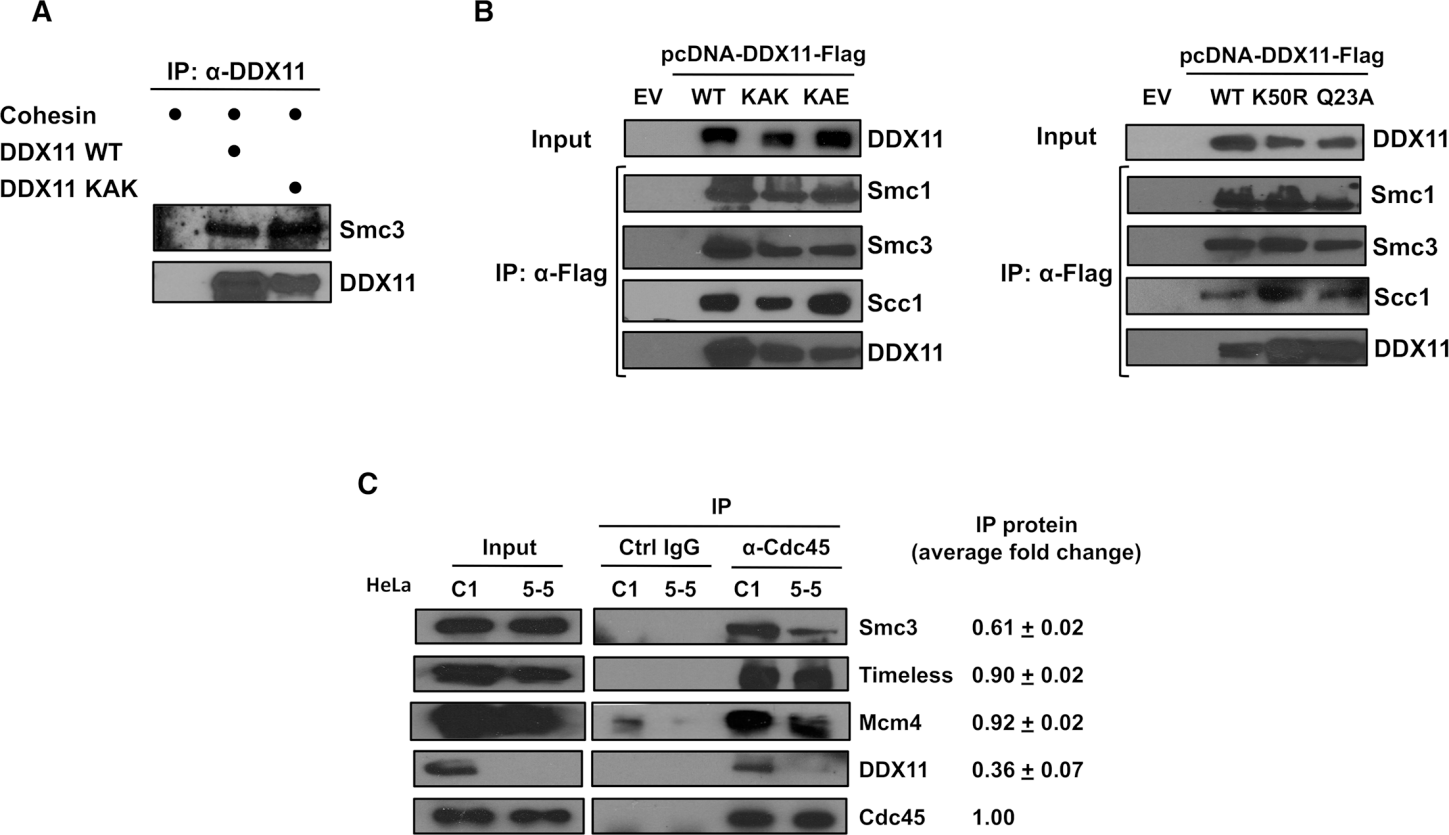
DDX11 interacts with the cohesin complex. ***A***) Purified recombinant DDX11 and cohesin directly interact. Co-pull down experiments on mixtures of the indicated purified recombinant proteins were carried out using an anti-DDX11 antibody bound to Protein A Sepharose beads. Pulled down samples were analysed by Western blot to detect the indicated proteins. ***B***) DDX11 interacts with the cohesin complex in cell extracts. IP experiments were carried out on extracts from HEK 293T cells transiently transfected with an empty vector (*EV*) and vectors over-expressing Flag-tagged wild type DDX11 (*WT*) and its mutant derivatives (*KAK* and *KAE*, on *left*; and *K50R* and *Q23A*, on *right*). Anti-Flag M2 agarose beads were used. ***C***) DDX11 and the cohesin complex are associated with the replication machinery on chromatin in S phase cells. IP experiments were carried out on the nuclear fraction of control (*C1*) and DDX11-downregulated (*5–5*) HeLa cells with control and anti-Cdc45 rabbit IgG bound to Protein A Sepharose beads. The immuno-precipitated samples were analyzed by Western blot to detect the indicated proteins. Experiments were carried out in triplicate and level of the immuno-precipitated proteins (median values with standard errors) are indicated. Reported values were normalized to the level of Cdc45 pulled down in each sample. Quantitative analyses of immuno-blot signals were carried out using the ImageJ software.

Then, we examined if DDX11 was able to interact with cohesin. To this end, we carried out co-pull down experiments using purified recombinant proteins. Flag-tagged DDX11 wild type and KAK mutant were purified from transiently transfected mammalian cells. Human cohesin was produced in insect cells infected with a single multi-gene baculovirus co-expressing the four core complex subunits (Smc1, Smc3-Flag, Scc1, 10xHis-SA1; S7 Fig). Co-pull down experiments with an anti-DDX11 antibody bound to Protein A Sepharose beads revealed that either wild type DDX11 or the KAK mutant was able to bind the cohesin complex (Fig 5A). Besides, we carried out co-immunoprecipitation experiments with anti-Flag beads on extracts of HEK 293T cells transiently transfected with vectors expressing a Flag-tagged DDX11 wild type and its mutants, and found that endogenous cohesin was co-pulled down with each of these proteins (Fig 5B). These findings suggest that DDX11 physically associates with cohesin, and this interaction does not require the "EYE" motif or the helicase activity of DDX11.

Chl1, the budding yeast counterpart of human DDX11, was reported to engage with cohesin during S phase in the context of the replication fork [36]. To examine whether human DDX11 and cohesin associate with the advancing replisomes, we carried out co-immuno-precipitation experiments with an anti-Cdc45 antibody bound to Protein A Sepharose beads on the nuclear fraction of control (HeLa C1) and DDX11-depleted HeLa cells (HeLa 5–5). Cdc45 is an accessory subunit of the replicative DNA helicase (the *C*dc45/*M*cm2-7/*G*INS, CMG, complex) [37]. As shown in Fig 5C, Western blot analysis of the pulled down samples revealed the association to Cdc45 of DDX11, cohesin (Smc3 subunit; binding of Smc1 is shown in S8A Fig), Timeless and the Mcm4 protein. Depletion of DDX11 reduced the amount of cohesin that was co-immunoprecipitated with Cdc45, but had no effect on the pull-down of Mcm4 and Timeless (Figs 4C and S8A). Since Cdc45 associates with chromatin only in S phase as a stable component of the CMG complex together with GINS [37], our results suggest that DDX11 is bound to the advancing replisomes and plays a critical role in anchoring cohesin to the replication forks.

**Fig 6:**
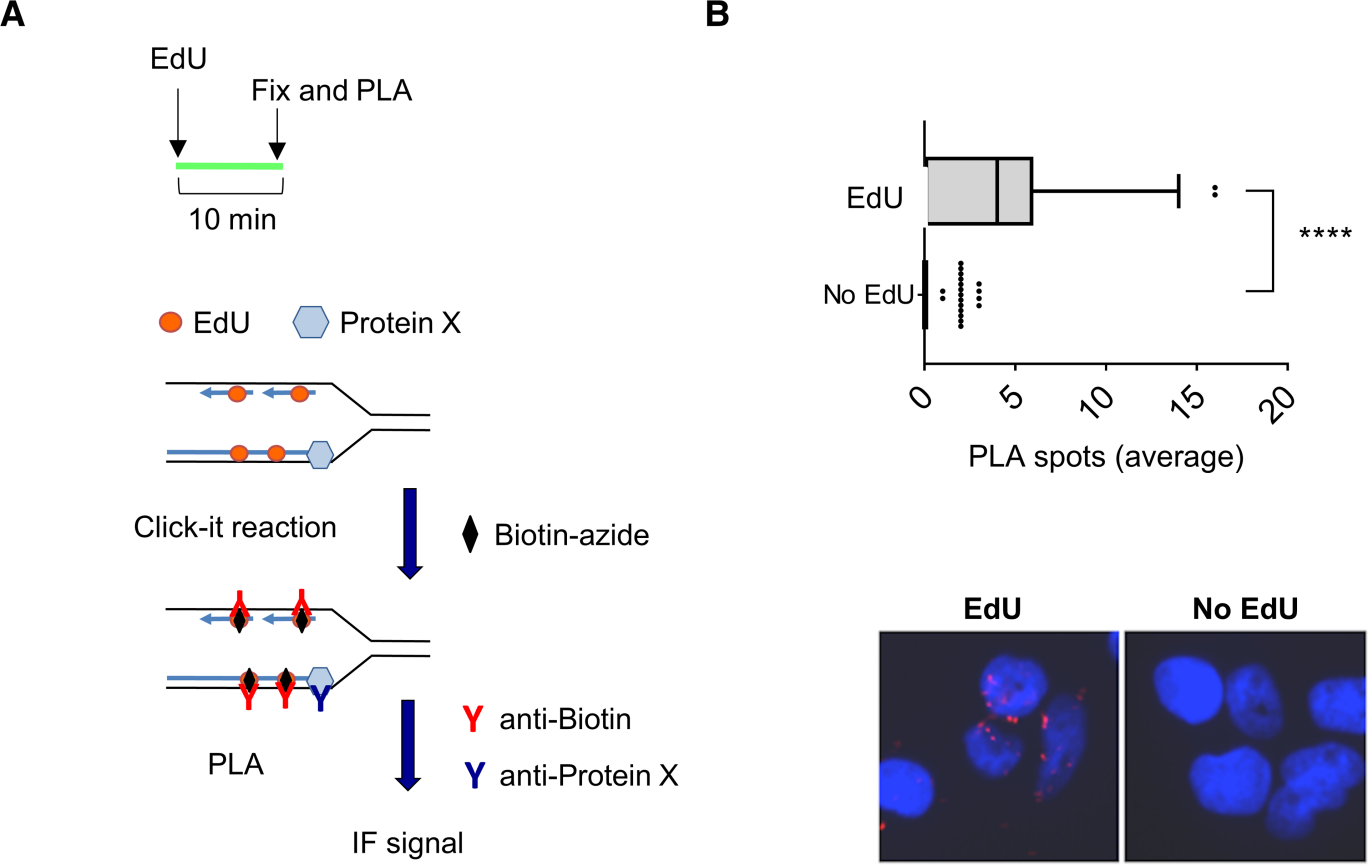
DDX11 localises at DNA replication forks. ***A***) Experimental scheme of SIRF assay. Cells were pulsed with EdU for 10 min. Incorporated EdU was biotinylated using click chemistry. Then, cells were fixed and PLA was performed using anti-biotin and anti-DDX11 antibodies. Unlabelled controls were subjected to PLA (*no EdU*). ***B***) The graph shows the mean number of PLA spots per cell. Values are presented as means ± standard errors (**** *P* < 0.0001; Anova test). Representative images are shown.

While evidences of an association of cohesin and many cohesin regulators (such as Esco2, the Scc2-Scc4 loader and the Wapl-Pds5 releasing complex) to the replication machinery were reported by various experimental approaches in mammalian cells [35, 38–40], localisation of DDX11 at sites of DNA synthesis has remained elusive so far. To further investigate this issue, we used the *in* *s**itu* analysis of protein *i*nteractions at DNA *r*eplication *f*orks (SIRF) technique [41]. As schematically depicted in Fig 6A, this novel methodology is based on a *p*roximity *l*igation *a*ssay (PLA) coupled to 5'-ethylene-2'-deoxyuridine (EdU) click-it chemistry to identify co-localisation of proteins of interest to nascent DNA in single cells. This analysis revealed that the number of DDX11-EdU PLA spots was significantly higher in EdU-treated cells than in control cells (see Fig 6B), indicating that DDX11 localises at sites of DNA synthesis.

Finally, we carried out an additional set of co-immunoprecipitation experiments on extracts of HEK 293T transiently expressing Flag-tagged DDX11 wild type and mutant proteins and found that association of DDX11 KAE and KAK mutants to various components of the DNA replication machinery (such as Timeless, WDHD1 and Cdc45) was noticeably reduced relatively to the wild type protein (S8B Fig). In contrast, binding of the DDX11 helicase-dead mutants (K50R and Q23A) to the above replication factors was similar to that of the wild type protein (see S8C Fig). These results suggest that DDX11 acts in concert with Timeless to ensure stable binding of cohesin to the advancing replisomes.

## Discussion

In this study we identified a sequence motif of the human DDX11 DNA helicase that is responsible for the direct interaction with Timeless, a component of the replication fork-protection complex. Using Timeless-binding defective and helicase-dead mutants of DDX11 in complementation studies, we were able to demonstrate that the interaction of DDX11 with Timeless is more critical for sister chromatid cohesion than its DNA helicase activity. Besides, we found that DDX11 and cohesin associate with the ongoing replication forks in S phase synchronized HeLa cells, and binding of cohesin to the replisomes is reduced when DDX11 is down-regulated. These results, together with our finding that DDX11 physically interacts with the cohesin complex *in vitro*, suggests that DDX11 might play a role in recruiting cohesin to the ongoing replication forks.

The short sequence motif of human DDX11 involved in Timeless-binding (the so-called "EYE" motif) maps to a 160-residue insertion between helicase boxes I and Ia, which we named Region T. A multiple sequence alignment of human SF2 Fe-S cluster DNA helicases revealed that XPD and RTEL1 do not have a corresponding N-terminal extra-domain. Human FANCJ has a shorter N-terminal insertion between helicase boxes I and Ia (S1 Fig), which was reported to be critical for binding either to the DNA mismatch repair protein MLH1 [42] or to G-quadruplex DNA structures [43].

The "EYE" motif is invariably present in DDX11 orthologs from vertebrates, but it is only partially conserved in the *Saccharomyces cerevisiae* Chl1 DNA helicase (see S3A and S3B Fig). Consistently, Chl1 was reported to be recruited to the replication forks by the homo-trimeric Ctf4 replication factor. This interaction is mediated by a specific *C*tf4-*i*nteracting *p*rotein (CIP) motif that maps in the C-terminal portion of the Chl1 protein between helicase boxes IV and V [36], as shown in S3A Fig. Similar CIP motifs were demonstrated to mediate the interaction of DNA polymerase α (*via* the p180 subunit) and the GINS complex (*via* the Sld5 subunit) with Ctf4 factor [44]. Multiple sequence alignments revealed that DDX11 vertebrate orthologs lack a conserved CIP motif between helicase boxes IV and V (S3C Fig). Overall, these findings suggest that the network of interactions among protein factors operating at the DNA replication fork is not completely conserved during evolution from yeasts to vertebrates.

Our analysis further revealed that two DDX11 site-specific mutants devoid of ATPase and DNA helicase activities (DDX11 K50R and Q23A) were able to partially rescue sister chromatid cohesion defects of DDX11-depleted HeLa cells. In contrast, in avian DDX11-knocked-out DT40 cells DDX11 helicase-dead mutant (K87A, chicken counterpart of human DDX11 K50R mutant) was unable to rescue the defective sister chromatid cohesion phenotype in complementation assays [45]. However, our finding, that the enzymatic activities of human DDX11 are not strictly required for sister chromatid cohesion, is in agreement with a published study on the budding yeast Chl1 [36]. An important implication of our results is that, even if Chl1/DDX11 DNA helicase activity is involved in Okazaki fragment processing in yeast/human cells, as previously hypothesized [10, 12], this function is not required for efficient chromosomal cohesion. This hypothesis is consistent with a recent biochemical study revealing that DNA-bound fission yeast cohesin can capture a second DNA molecule only if that is single-stranded. Thus, second-DNA capture by the cohesin ring is likely to occur at the replication fork prior to Okazaki fragment maturation by a process that is ATP-dependent and strictly requires the Scc2-Scc4 loader [46].

It was proposed that the budding yeast Chl1 protein could also promote cohesion establishment at the replication fork by orienting the cohesin complex in a way that facilitates acetylation of the Smc3 subunit by the specific Eco1 acetyltransferase [36]. Indeed, a remarkable reduction of Smc3 acetylation was observed in yeast cells lacking the *Chl1* gene [15]. We found that down-regulation of DDX11 did not substantially affect the level of acetylated Smc3 in HeLa cells (see S4C Fig), in agreement with an analysis carried out in chicken DDX11-knockout DT40 cells [45]. These results suggest that other protein factors assist Esco1/Esco2 in modifying cohesin during cohesion establishment at the replication fork in mammalian cells. Indeed, a recent study by Peters and coworkers has shown that Esco2 directly interacts with the MCM2–7 complex in human cells and this interaction is critical for cohesin acetylation and cohesion establishment [40].

In this study we provide evidence that DDX11 is located at sites of DNA synthesis in single cells by using the SIRF technique [41]. Moreover, our analysis suggests that DDX11 is involved in anchoring the cohesin complex to the replication machinery during fork progression. This hypothesis is supported by our finding that DDX11 physically interacts with cohesin either in cell extracts or *in vitro* using purified recombinant proteins. The association between cohesin and DDX11 takes place in the context of the ongoing DNA replication forks, as suggested by their co-immunoprecipitation from the nucleoplasm/chromatin fraction of HeLa cell extracts with an antibody specific for Cdc45, a component of the CMG complex that is bound to replisomes only during S phase [37]. Moreover, we found that depletion of DDX11 in HeLa cells reduces the amount of cohesin that is co-pulled down with Cdc45. Besides, in cells over-expressing DDX11 mutants with impaired Timeless-binding capability we found a reduced association of either DDX11 or cohesin to the DNA replication machinery (Figs 5 and S8). Our results and reports by others [19] suggest that Timeless may act upstream of DDX11 to enable a stable association of cohesin rings to the DNA replication forks. However, we cannot exclude that additional replication factors help to stabilize association of cohesin to the replication machinery. In fact, a comprehensive parallel study suggests that cohesin is loaded at the pre-replication complex at the G1/S boundary in a process that requires Scc2-Scc4, the Mcm2-7 complex and the *D*bf4-*d*ependent *k*inase (DDK); after replication origin firing and Mcm2-7 DNA helicase activation, cohesin rings are mobilized and held at ongoing replication forks with the help of other replication factors, in addition to Timeless and DDX11, including WDHD1 and RPA [35].

In a previous work we provided evidence that DDX11 and Timeless directly interact and operate in the same pathway that preserves replication fork progression in stressful conditions (such as dNTP depletion) [28]. In the present work, we found that the DDX11-Timeless interaction is critical for establishing chromosomal cohesion, whereas the enzymatic activities of DDX11 are not as essential for this process. This finding suggests that restarting stalled replication forks in stressful conditions and pairing duplicated chromatids are separable functions of DDX11, even if the interaction with Timeless is needed to efficiently execute both.

Our results have important implications for understanding the molecular basis of WABS. The few pathogenic missense mutations described so far were demonstrated to abolish the DDX11 DNA helicase activity *in vitro* by biochemical studies [26–27]. However, in fibroblasts derived from WABS patients, an almost complete disappearance of DDX11 was observed by immuno-blot analysis [26], quite likely due to an intrinsic instability of the mutated protein. Thus, in line with our findings, it is likely that the chromosomal cohesion defects observed in WABS patient-derived cell lines are caused by DDX11 protein loss rather than by abrogation of its catalytic activities.

## Materials and methods

### Plasmid construction, protein expression and purification

Recombinant human Flag-Timeless was produced in insect cells infected with a recombinant baculovirus, as previously described [47]. DDX11-3xFlag (wild type and KAK mutant) were produced in HEK 293T cells transiently transfected with pcDNA 3.0 plasmid constructions and purified as previously described [33]. The cohesin complex (consisting of Smc1, Smc3-Flag, Scc1, 10xHis-SA1) was produced in *Sf9* insect cells infected with a single multi-gene recombinant baculovirus (a gift from Jan-Michael Peters, Wien, Austria), as previously described [48].

### Protein interaction studies using peptide microarrays

Customized PEPperCHIP peptide microarrays were provided by PEPperPRINT GmbH (Heidelberg, Germany) on conventional glass slides (75.4 mm x 25 mm x 1 mm). In the tiling peptide microarray DDX11 protein was translated into 454 different 15-amino acid peptides with a peptide-peptide overlap of 13 residues and spotted in duplicate (908 peptide spots for each array). In the microarray containing a full substitution scan of DDX11 peptide # 32 (NH_2_-EQLESGEEELVLAEY-COOH), all residues of this peptide were substituted with all 20 natural amino acids (300 spots containing 15-amino acid peptides). Moreover, each microarray was framed by Flag (NH_2_-DYKDDDDKAS-COOH) and HA (NH_2_-YPYDVPDYAG-COOH) tags as control peptides. Each peptide microarray was incubated using a PEPperCHIP incubation tray (PEPperPRINT, Heidelberg, Germany) with shaking at 140 rpm in the following solutions in sequence: blocking buffer (PBS containing 0.05% [v:v] Tween 20, 1% [w:v] bovine serum albumin) 1 hr at room temperature; staining buffer (PBS containing 0.05% [v:v] Tween 20, 0.1% [w:v] bovine serum albumin) for 10 min; blocking buffer containing Flag-Timeless at 50 μg/mL for 16 hr; washing buffer (PBS containing 0.05% [v:v] Tween 20) for 5 s at room temperature for three times; blocking buffer containing Cy3-labelled anti-Flag and Cy5-labelled anti-HA antibodies for 30 min at room temperature; washing buffer for 5 s at room temperature for three times. The chip was dipped into de-ionized water, dried in a stream of air and analyzed in a high-resolution fluorescence microarray scanner (Agilent, model G2565).

### Antibodies

The following primary antibodies were used for immunoblotting, immunoprecipitations and immunofluorescence to detect human proteins of interest: Timeless (Abcam, ab72458); DDX11 (Santacruz Biotechnology, sc-271711; for the SIRF assay, a rabbit polyclonal antibody donated by Joanna Parish, Birmingham, United Kingdom); CREST (donated by Florence Larminat, Toulouse, France); Tubulin (Sigma, T9026); Smc3 (Bethyl, A300-060A); GAPDH (Cell Signalling, 2118S); Smc1 (Abcam, ab117610); Scc1 (Cell Signaling Technology, 4321S); Mcm4 (Abcam, ab4459); Cdc45 (a rat monoclonal antibody donated by Hans-Peter Nasheuer, Galway, Ireland; our own rabbit polyclonal against full length Cdc45 used in the immuno-precipitation experiments of Figs 5C and S8A). In addition, the following antibodies specific for protein tags were used: Flag (Abcam, ab49763; Pepperprint, Cy3-labelled antibody 110802); HA (Pepperprint, Cy5-labelled antibody 110801); Myc (Roche, 11667203001). A mouse monoclonal anti-biotin (Invitrogen, 03–3700) was used for the SIRF assay. The following horseradish peroxidase-conjugated secondary antibodies were used: anti-rabbit (Abcam, ab6721); anti-mouse (Santacruz Biotechnology, sc-2005); anti-rat (Sigma, A5795).

### Cell lines

A DDX11-defective HeLa cell line (named HeLa 5–5), was kindly provided by Dr Akira Inoue (Memphis, TN, USA). It was established by infection of HeLa cells with a pantropic retrovirus (pSuper-Retro-Puro) expressing a shRNA (# 5, targeting the DDX11 coding sequence, U33833, from nucleotide 2398), together with an HeLa control clone (named HeLa C1), obtained by infection with an empty retrovirus construction, as previously described [30–31]. HeLa and human embryonic kidney (HEK) 293T cells were cultured in Dulbecco's modified Eagle's medium supplemented with 10% fetal bovin serum (FBS) and Pen/Strep in a humidified 5% CO_2_ atmosphere at 37°C.

### Co-immunoprecipitation experiments from whole cell extract

pcDNA 3.0 plasmids were constructed that direct over-expression of the DDX11 protein fused to a 3x Flag-tag at the C-terminal end (wild type and site-specific mutants). These plasmid vectors were transfected into HEK 293T cells using PEI at a mass ratio of 3:1 PEI:DNA. At 48 hr after transfection, cells (about 1 x 10^8^ cells/experiment) were detached, washed twice in cold PBS. Cell pellets were re-suspended in lysis buffer (50 mM Tris-HCl pH 8.0, 150 mM NaCl, 0.25% [v:v] Triton X-100, 10% [v:v] glycerol) supplemented with a protease inhibitor cocktail. The samples were subjected to sonication on ice using a Branson digital sonifier model SSE-1 (8 cycles consisting of 2-s impulses at an output 10% followed by 5-s intervals) and centrifuged for 10 min at 13,000 *g* at 4°C. Then, 30 μL of Flag-M2 (Sigma) beads were added to 2 mg of cell extract total protein. Samples were incubated at 4°C for 2 hr in a rotating wheel. Then, beads were washed four times with lysis buffer and proteins and protein complexes specifically bound were eluted with lysis buffer containing Flag peptide at 0.4 mg/mL. Samples were subjected to Western blot analysis using the indicated antibodies.

### Co-immunoprecipitation experiments from cell nuclear fraction

Immuno-precipitations were carried out on nuclear extracts prepared from the indicated HeLa cells (about 4 x 10^7^ cells/experiment). Cell cultures were synchronized in S phase with a single block in thymidine (at 2 mM) followed by release in fresh medium for 2.5 hr. Cells were collected by centrifugation. Preparation of cell nuclear fraction was according to a published protocol with modifications [49]. Cell pellets were re-suspended in 1 mL of osmotic buffer (10 mM Hepes-NaOH pH 7.9, 0.2 M potassium acetate, 0.34 M sucrose, 10% [v:v] glycerol, 1 mM dithiotreitol, 0.1% [v:v] Triton X-100) and incubated for 5 min on ice. After centrifugation (800 *g* for 5 min), the nucleus/chromatin fraction present in the pellet was re-suspended in 1 mL of hypotonic buffer (10 mM Hepes-NaOH pH 7.9, 50 mM NaCl, 1 mM dithiotreitol, 0.1% [v:v] Triton X-100) containing a protease inhibitor cocktail (Roche). Samples were subjected to sonication on ice using a Branson digital sonifier model SSE-1 (10 cycles consisting of 10-s impulses at an output 10% followed by 20-s intervals) followed by incubation for 20 min at 37°C in the presence of micrococcal nuclease (2 units/sample; Sigma, cat. N3755) and CaCl_2_ (at 10 mM). Insoluble material was removed by centrifugation at 16,000 *g* for 30 min. Samples (containing 0.3–0.5 mg of protein) were used in immuno-precipitation experiments with the indicated rabbit antibodies and control rabbit IgG bound to Protein A Sepharose beads (GE Healthcare). They were incubated for a minimum of 3 hr (or overnight) at 4°C in a rotating wheel. Beads were washed 4 times with the following buffer: 10 mM Hepes-NaOH pH 7.9, 50 mM NaCl, 1 mM dithiotreitol, 0.1% (v:v) Triton X-100. Proteins bound to the beads were re-suspended in SDS-PAGE loading buffer (50 mM Tris-HCl, pH 6.8, 10% [v:v] glycerol, 200 mM β-mercaptoethanol, 0.5% [w:v] SDS, 0.01% [w:v] blue bromophenol) and analyzed by Western blot using the indicated antibodies.

### *In vitro* pull down assays

Direct interaction between recombinant purified Timeless (or cohesin) and DDX11 proteins was analyzed by co-immuno-precipitation experiments. Mixtures (200 μL) contained purified Timeless (0.8 μg) or the cohesin complex, (1 μg), recombinant DDX11 (0.5 μg) and 30 μL of Protein A-beads bound to anti-DDX11 antibody in binding buffer (25 mM Tris-HCl, pH 7.5, 150 mM NaCl, 2 mM MgCl_2_,1 mM dithiotreitol, 5% [v:v] glycerol). Samples were incubated for 2 hr at 4°C on a rotating wheel. Then, the beads were washed 4 times with washing buffer (25 mM Tris-HCl, pH 7.5, 300 mM NaCl, 2 mM MgCl_2_,1 mM dithiotreitol, 5% [v:v] glycerol, 0.25% [v:v] Triton X-100) and bound proteins re-suspended in 30 μL of SDS-PAGE loading buffer. Samples were subjected to electrophoresis through 7% polyacrylamide-bis (29:1) gel and analyzed by immuno-blot with the indicated antibodies.

### Analysis of metaphase chromosome spreads

Plasmid constructs expressing wild type DDX11 and its indicated site-specific mutants were transfected into DDX11-depleted HeLa cells (clone 5–5; [30–31]). pcDNA 3.0 vector constructs (named pcDNA-DDX11-Flag_WT, _K50R, _Q23A, _KAK and _KAE) were mutated to make the DDX11 coding sequence resistant to the short hairpin RNA # 5 that is stably produced in the above HeLa cell line to down-regulate the endogenous DDX11 expression. At 24 hr post-transfection, cells were blocked in S phase by adding thymidine at 2 mM into the medium. After 16 hr, cells were released into fresh medium without thymidine. After 9 hr, colchicine at 5 μM was added to the medium and cultures incubated for additional 2 hr. Then, mitotic cells were collected by shake-off, washed once with PBS, treated with 55 mM KCl hypotonic solution at 37°C for 15 min and spun onto microscope slides with a Shandon Cytospin centrifuge. Cells on the slides were first treated with the PHEM buffer (25 mM HEPES, pH 7.5, 10 mM EGTA, pH 8.0, 60 mM PIPES, pH 7.0, and 2 mM MgCl_2_) containing 0.3% (v:v) Triton X-100 for 5 min and then fixed in 4% (v:v) paraformaldehyde for 10 min. Fixed cells were washed three times with PBS containing 0.1% (v:v) Triton X-100 for 2 min each time, and incubated with the human CREST antiserum in PBS containing 3% (w:v) BSA and 0.1% (v:v) Triton X-100 at 4°C overnight. Cells were then washed three times with PBS containing 0.1% (v:v) Triton X-100 for 2 min each time, and incubated with a fluorescent secondary antibody in PBS containing 3% (w:v) BSA and 0.1% (v:v) Triton X-100 for 1 hr at room temperature. Cells were again washed three times with PBS containing 0.1% (v:v) Triton X-100 and then stained with 1 μg/mL DAPI for 2 min. Slides were viewed with a 100x objective on a Nikon A1 confocal microscope using a NIS-Elements imaging software.

### Fluorescence *in situ* hybridization (FISH) analysis

FISH probes that specifically recognize a locus on human chromosome 3 were made as described previously [50]. HeLa cells expressing shCtrl (HeLa C1) or shDDX11 (HeLa 5–5) were transfected with plasmid vectors that express wild type or mutant DDX11. After a thymidine-block for 16–18 hr, cultures were released to fresh medium and incubated for 4 hr. Then, cells were harvested by treatment with Trypsin, treated with 75 mM KCl hypotonic solution for 25 min at 37°C and fixed with ice-cold methanol and acetic acid (3:1, v:v). Fixed cells were dropped onto pre-warmed slides, *in situ* hybridized at 80°C with DNA probes and incubated at 37°C overnight. Slides were sequentially washed with 0.1% (w:v) SDS in 0.5x SSC at 70°C for 5 min, PBS at room temperature for 10 min and 0.1% (v:v) Tween 20 in PBS at room temperature for 10 min. Slides were then mounted with ProLong Gold (Life Technologies) and viewed with a 100 x objective on a DeltaVision fluorescence microscope (GE Healthcare). Image processing and quantification were performed with the ImageJ software.

### Immunofluorescence analysis of HeLa cells

For siRNA transfection, HeLa Tet-On cells at 20–40% confluency were transfected with Lipofectamine RNAiMAX (Invitrogen) according to the manufacturer’s protocols, and analyzed at 24–48 hr after transfection. The siRNAs were transfected at a final concentration of 5 nM. The siRNAs used to downregulate DDX11 expression were as follows: # 1 (5'-GCAGAGCUGUACCGGGUUU-3'), # 2: (5'CGGCAGAACCUUUGUGUAA-3'), # 3: (5'-GAGGAAGAACACAUAACUA-3'), # 4: (5'-UGUUCAAGGUGCAGCGAUA-3'). Cells were cultured and treated in the Nunc Lab-Tek II CC2 Chamber Slides. They were first treated with the PHEM buffer containing 0.5% (v:v) Triton X-100 for 5 min and then fixed in 2% (v:v) paraformaldehyde for 15 min. Fixed cells were blocked in PBS containing 2% (w:v) BSA for 30 min and then incubated with desired antibodies in PBS containing 0.1% (v:v) Triton X-100 (PBST) and 3% (w:v) BSA and at 4°C overnight. Cells were then washed three times with PBST for 5 min each time, and incubated with fluorescent secondary antibodies (Molecular Probes) in PBST containing 3% (w:v) BSA for 1 hr at room temperature. Cells were again washed three times with PBST and stained with 1 μg/mL DAPI in PBS for 5 min. After the final wash with PBS, the slides were mounted with VECTASHIELD anti-fade mounting medium (Vector Laboratories), sealed with nail polish, and viewed with a 100x objective on a DeltaVision fluorescence microscope (GE Healthcare). Image processing and quantification were performed with Image J.

### SIRF assay

Exponential growing MRC5SV40 cells (described in [51]) were seeded onto a microscope chamber slide. The day of experiment, cells were incubated with 100 μM EdU for 15 min and treated as indicated. After treatments cells were pre-extracted in CSK-100 buffer (100mM NaCl, 300mM sucrose, 3 mM MgCl_2,_10 mM PIPES pH 6.8, 1 mM EGTA, 0.2% Triton X-100, protease inhibitor cocktail at 1x) for 5 min on ice under gentle agitation and fixed with 4% (v:v) paraformaldehyde in PBS for 20 min at RT. Cells were treated with ice-cold methanol at -20°C for 10 s and then blocked in 3% (w:v) BSA in PBS for 15 min. The primary antibodies used were diluted as follows: rabbit anti-DDX11 at 1:150 and mouse anti-biotin at 1:500. The negative control consisted of cells that were not pulsed with EdU. Samples were incubated with secondary antibodies (OLINK Bioscience) conjugated with PLA probes MINUS (anti-rabbit) and PLUS (anti-mouse). The incubation with all antibodies was accomplished in a humidified chamber for 1 h at 37°C. Next, the PLA probes MINUS and PLUS were hybridized to two connecting oligonucleotides to produce a template for rolling-circle amplification. After amplification, the products were hybridized with a red fluorescence-labelled oligonucleotide. Samples were mounted in Prolong Gold anti-fade reagent with DAPI. Images were acquired randomly using an Eclipse 80i Nikon fluorescence microscope, equipped with a Video Confocal (ViCo) system.

## Supporting information

S1 Fig Identification of modular insertions in human SF2 Fe-S cluster containing DNA helicases. Schematic representation of the indicated human DNA helicases polypeptide chain. The conserved helicase boxes (from I to VI) are indicated. Sequence motifs interacting with the indicated proteins are indicated with different colours. The abbreviations used are: *Q*, for Q motif; *Fe-S*, for Fe-S cluster; *Arch*, for Arch domain; *PIP*, for PCNA-interacting protein motif; *BLM*, for Bloom helicase; *Tim*, for Timeless. N-terminal and C-terminal insertions are indicated in *green* and in *red*, respectively. A drawing schematically showing the insertions in the putative three-dimensional structure of each DNA helicase is shown on *right* (modified from [43]).
(TIFF)

S2 Fig Identification of DDX11 amino acid residues critical for Timeless-binding. Microarrays containing a full substitution scan of peptide # 32 were probed with (on *left*) or without (control, on *right*) purified recombinant Flag-tagged Timeless and detected with a mixture Cy3-labelled anti-Flag antibody and Cy5-labelled anti-HA antibody. Images of microarrays analyzed with a high-resolution fluorescence scanner are shown. The sequence of DDX11 peptide # 32 is reported on the *top* of each microarray; amino acid changes in each row of the array are reported on *right*. Amino acid substitutions that reduce or abolish the interaction with Timeless are highlighted in *red*.
(TIFF)

S3 Fig ***A***) Schematic representation of the polypeptide chain of *Homo sapiens* (*Hsa*) DDX11 and *Saccharomyces cerevisiae* (*Sce*) Chl1, both belonging to the group of SF2 DNA helicases with a Fe-S cluster. Conserved helicase motifs (from I to VI) are indicated in *red*. Other sequence motifs are indicated with different colours. Abbreviations used are: *Q*, for Q motif; *Fe-S*, for Fe-S cluster; *Arch*, for Arch domain; *CIP*, for *C*tf4-*i*nteracting *p*rotein motif; *T*, for Timeless-interacting Region T. ***B***) Multiple alignment of the putative DDX11/Chl1 "EYE" motif from various organisms. Amino acids shown to be essential for interaction with Timeless in human DDX11 are in *red*. ***C***) Alignment of putative CIP motifs of various yeast and human proteins and their partial conservation in vertebrate DDX11 orthologs. *Pol1*_*YEAST* stands for *Saccharomyces cerevisiae* DNA polymerase 1 catalytic subunit; *Pol α_HUMAN* stands for *Homo sapiens* DNA polymerase α p180 subunit. Amino acids shown to be essential for interaction with Ctf4 n budding yeast proteins are in *red* [36, 44]. Highly conserved residues in the aligned sequences are highlighted in *yellow*. In *B* and *C* the aligned sequences are from the following species: *Homo sapiens* (*HUMAN*), *Mus musculus* (*MOUSE*), *Meleagris gallopavo* (*TURKEY*), *Salmo salar* (*SALMON*), *Danio rerio* (*ZEBRAFISH*), *Xenopus laevis* (*XENOPUS*), *Drosophila melanogaster* (*FLY*), *Caenorhabditis elegans* (*WORM*), *Saccharomyces cerevisiae* (*YEAST*).
(TIFF)

S4 Fig ***A***) Analysis of metaphase chromosome spreads in DDX11/Wapl co-depleted HeLa cells. Representative images of HeLa cells with not separated or prematurely separated chromatids. Metaphase spreads were stained with DAPI (*blue*) and the kinetochore marker CREST (*red*). *Scale bar*, 5 μm. ***B***) Quantification of HeLa cells with premature chromatid separation. Cells were transfected with the indicated siRNAs and enriched in mitosis. Sequence of the siRNA used to downregulate Wapl was: 5'-CGGACTACCCTTAGCACAA-3'. ***C*)** Level of acetylated Smc3 in DDX11-depleted HeLa cells. Immuno-blot showing level of the indicated proteins in HeLa cells expressing DDX11-shRNA (*HeLa 5–5*) and control line (*HeLa C1*). Samples containing 4 μg (*lane 1*), 6 μg (*lane 2*) and 8 μg (*lane 3*) of total protein present in the cell extract nuclear fraction were employed for Western blot analyses. Mouse monoclonal antibodies against Smc3 acetylated peptide 97-SLRRVIGAK^Ac^K^Ac^DQYFLDKKMC-116 and against the same not-acetylated peptide (a gift of Katsuhiko Shirahige, Tokyo, Japan) were used for detection. All samples were run on the same gel and blots were cut horizontally. Blots used to detect acetylated Smc3 were stripped and subsequently probed for Smc3. The relative amount of acetylated Smc3 (normalized to total Smc3) in HeLa C1 and 5–5 cells was quantified by comparing chemiluminescent signal intensities using the program ImageJ. Analyses were carried out in triplicate and median values of Ac-Smc3/Smc3 were: 0.93 ± 0.07 (HeLa C1) and 0.86 ± 0.05 (HeLa 5–5).
(TIFF)

S5 Fig DDX11 promotes cohesin loading onto chromatin in S phase HeLa cells. ***A***) DAPI (*blue*) and anti-SA2 (*red*) staining of HeLa cells transfected with the indicated plasmids and siRNAs and arrested in early S phase with thymidine. *Scale bar*, 5 μm. ***B***) Quantification of the SA2 chromatin intensities of cells in *A*. Each dot in the graph represents a single cell. Mean values and standard deviations (Myc-vector/siLuc, n = 115; Myc-vector/siDDX11, n = 102; Myc-DDX11/siDDX11, n = 97; Myc-vector/siScc2, n = 84). According to Student’s *t*-test, a value of *P* < 0.0001 was calculated for Myc-vector/siDDX11 versus Myc-DDX11/siDDX11. ***C***) Extracts of HeLa cells transfected with the indicated plasmids and siRNAs were blotted with the indicated antibodies.
(TIFF)

S6 Fig Complementation assays of Scc1-loading defect in DDX11-depleted cells. ***A***) DAPI (*blue*) and anti-Myc (*red*) staining of HeLa cells that stably expressed Scc1-Myc. Cells were transfected with the indicated plasmids and siRNAs and arrested in early S phase with thymidine. *Scale bar*, 5 μm. ***B***) Quantification of the Scc1-Myc chromatin intensities of cells in *A*. Each *dot* in the graph represents a single cell. Mean values and standard deviations (Flag-vector/siLuc, n = 103; Flag-vector/siDDX11, n = 134; WT/siDDX11, n = 81; Q23A/siDDX11, n = 102; K50R/siDDX11, n = 111; KAE/siDDX11, n = 127; KAK/siDDX11, n = 106; Flag-vector/siScc2, n = 118). According to Student’s *t*-test, a value of *P* < 0.0001 was calculated for the following dataset pairs: Flag-vector/siDDX11 versus WT/siDDX11, Q23A/siDDX11, K50R/siDDX11; WT/siDDX11 versus Q23A/siDDX11, K50R/siDDX11, KAE/siDDX11, KAK/siDDX11; K50R/siDDX11 versus KAE/siDDX11; a value of *P* = 0.0003 for Q23A/siDDX11 versus KAE/siDDX11; a value of *P* = 0.0022 for Q23A/siDDX11 versus KAK/siDDX11; a value of *P* = 0.0008 for K50R/siRNA versus Q23A/siDDX11. Not significant *P* values were calculated for the following dataset pairs: Flag vector/siDDX11 versus KAE/siDDX11 (*P* = 0.2722), KAK/siDDX11 (*P* = 0.1916); Q23A/siDDX11 versus K50R/siDDX11 (*P* = 0.8920); KAE/siDDX11 versus KAK/siDDX11 (*P* = 0.7628). ***C***) Extracts of HeLa cells transfected with the indicated plasmids and siRNAs were probed with the indicated antibodies in Western blot experiments.
(TIFF)

S7 Fig Recombinant proteins used in the present study. ***A***) and ***B***) SDS-PAGE analysis of recombinant human Timeless, purified from baculovirus-infected *Sf9* insect cells; DDX11 (wild type and KAK mutant), purified from HEK 293T cells transiently transfected with pcDNA 3.0 vector derivatives; cohesin core complex, purified from baculovirus-infected *Sf9* cells. Purification procedures are described in the *Materials and Methods* section. *M* indicates lane containing protein markers. Western blot analysis of purified recombinant Timeless, DDX11 WT and KAK mutant (50 and 100 ng of each protein sample) and purified cohesin complex (250 ng) were carried out using the indicated antibodies. ***C***) Plot showing DNA helicase activity of DDX11 wild type and KAK mutant. Samples were incubated for 60 min at 37°C, as previously described [28]. A radio-labelled forked duplex DNA was used as substrate. Gels were analysed using a phosphorimaging system.
(TIFF)

S8 Fig ***A***) DDX11 and the cohesin complex are associated with the replication machinery on chromatin in S phase cells. IP experiments were carried out on the nuclear fraction of control (*C1*) and DDX11-downregulated (*5–5*) HeLa cells with control and anti-Cdc45 rabbit IgG bound to Protein A Sepharose beads. The immuno-precipitated samples were analyzed by Western blot to detect the indicated proteins. Experiments were carried out in duplicate and level of the immuno-precipitated proteins (median values with standard errors) are indicated. Reported values were normalized to the level of Cdc45 pulled down in each sample. Quantitative analyses of immuno-blot signals were carried out using the ImageJ software. ***B***) Interaction of DDX11 with replisome components in cell extracts. IP experiments were carried out on extracts from HEK 293T cells transiently transfected with an empty vector (*EV*) and vectors over-expressing Flag-tagged wild type DDX11 (*WT*) and its mutant derivatives (*KAK* and *KAE*, on *left*; and *K50R* and *Q23A*, on *right*). Anti-Flag M2 agarose beads were used.
(TIFF)
