# Supplementary figures and images for: Interaction of the Warsaw breakage syndrome DNA helicase DDX11 with the replication fork-protection factor Timeless promotes sister chromatid cohesion
Source: PLoS Genet. 2018 Oct 10;14(10):e1007622. doi: 10.1371/journal.pgen.1007622 (PMC6179184; doi:10.1371/journal.pgen.1007622)

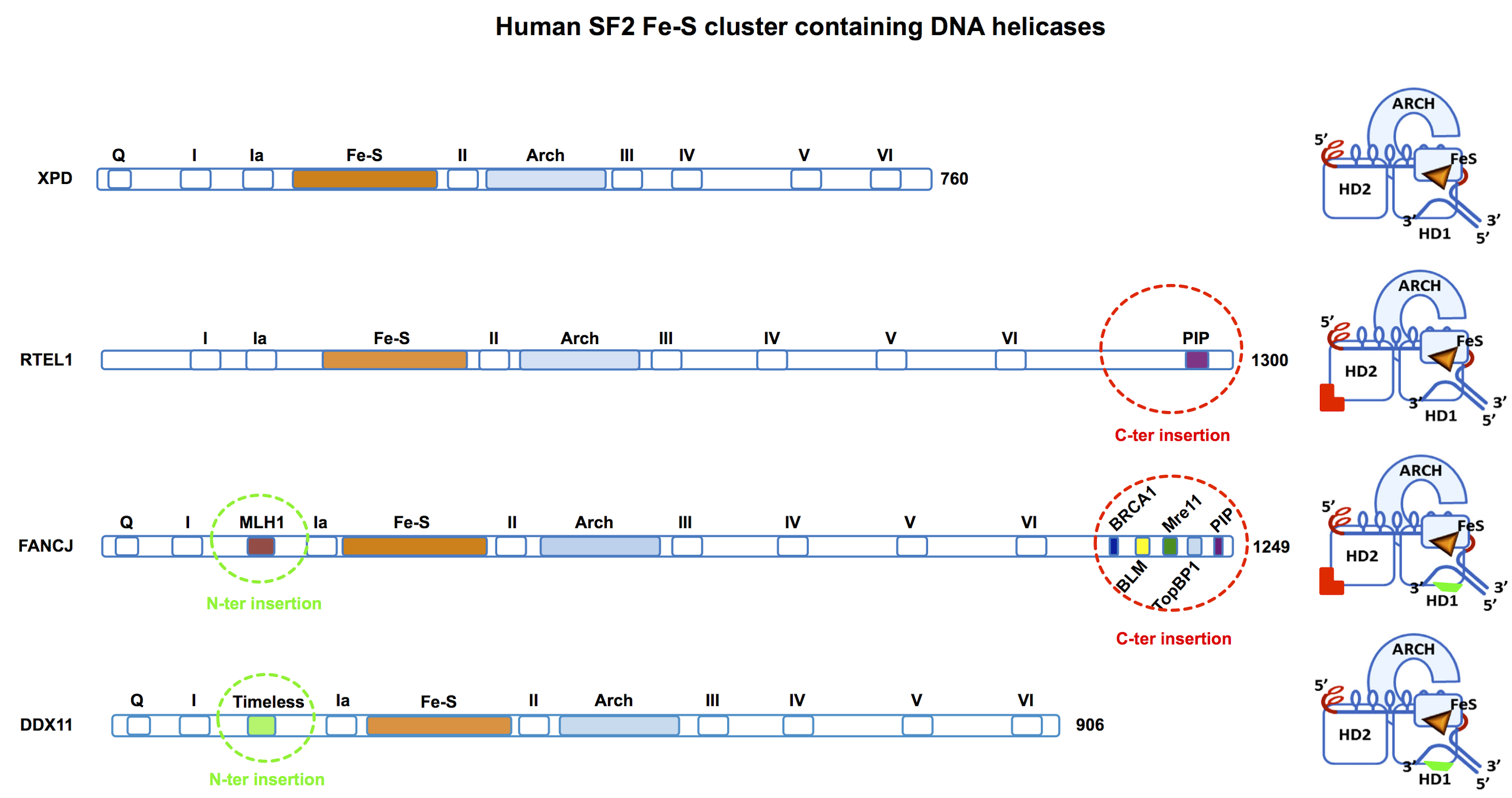

Supplement: S1 Fig — Schematic representation of the indicated human DNA helicases polypeptide chain. The conserved helicase boxes (from I to VI) are indicated. Sequence motifs interacting with the indicated proteins are indicated with different colours. The abbreviations used are: Q, for Q motif; Fe-S, for Fe-S cluster; Arch, for Arch domain; PIP, for PCNA-interacting protein motif; BLM, for Bloom helicase; Tim, for Timeless. N-terminal and C-terminal insertions are indicated in green and in red, respectively. A drawing schematically showing the insertions in the putative three-dimensional structure of each DNA helicase is shown on right (modified from [43]). (TIFF) [file pgen.1007622.s001.tiff]

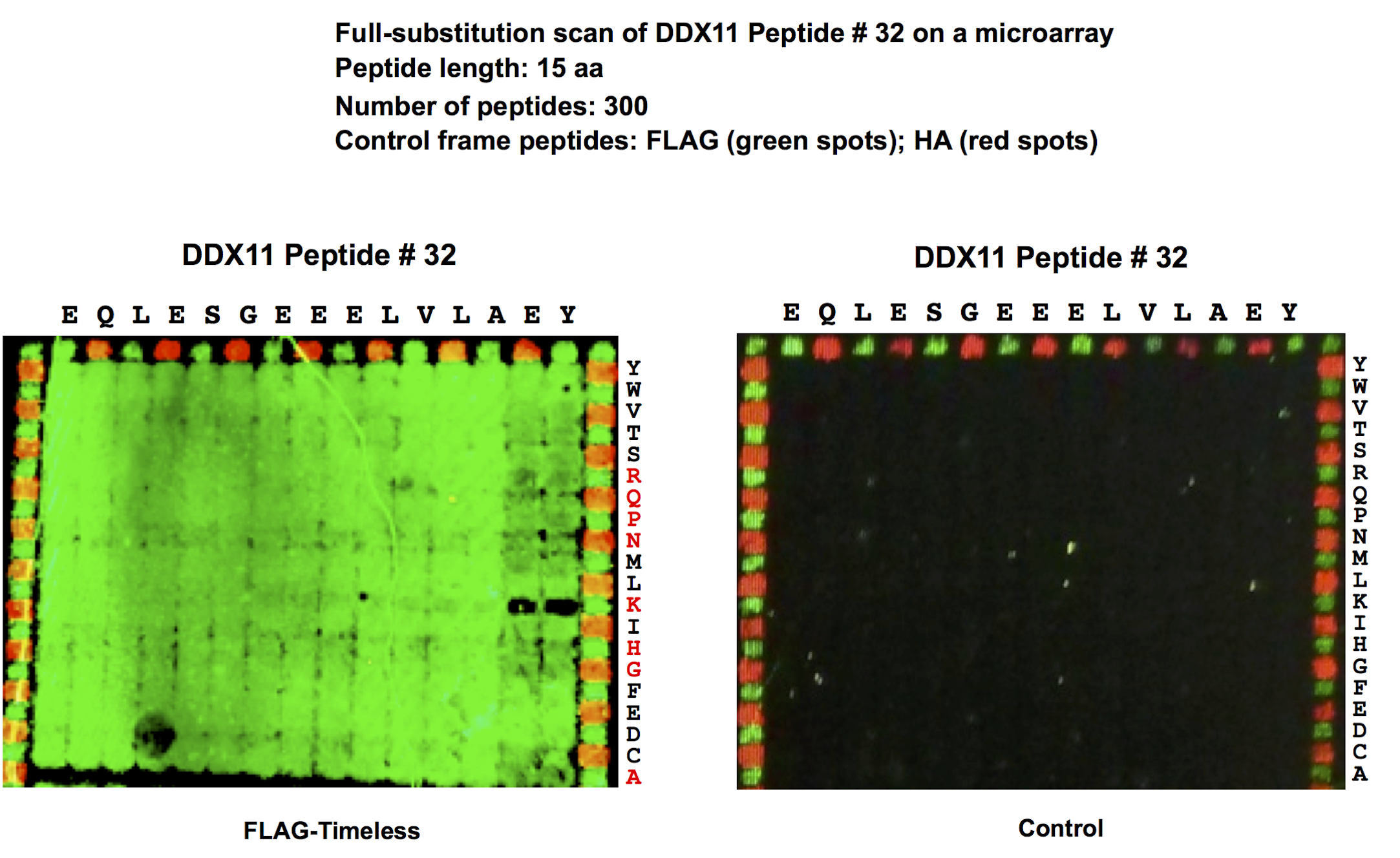

Supplement: S2 Fig — Microarrays containing a full substitution scan of peptide # 32 were probed with (on left) or without (control, on right) purified recombinant Flag-tagged Timeless and detected with a mixture Cy3-labelled anti-Flag antibody and Cy5-labelled anti-HA antibody. Images of microarrays analyzed with a high-resolution fluorescence scanner are shown. The sequence of DDX11 peptide # 32 is reported on the top of each microarray; amino acid changes in each row of the array are reported on right. Amino acid substitutions that reduce or abolish the interaction with Timeless are highlighted in red. (TIFF) [file pgen.1007622.s002.tiff]

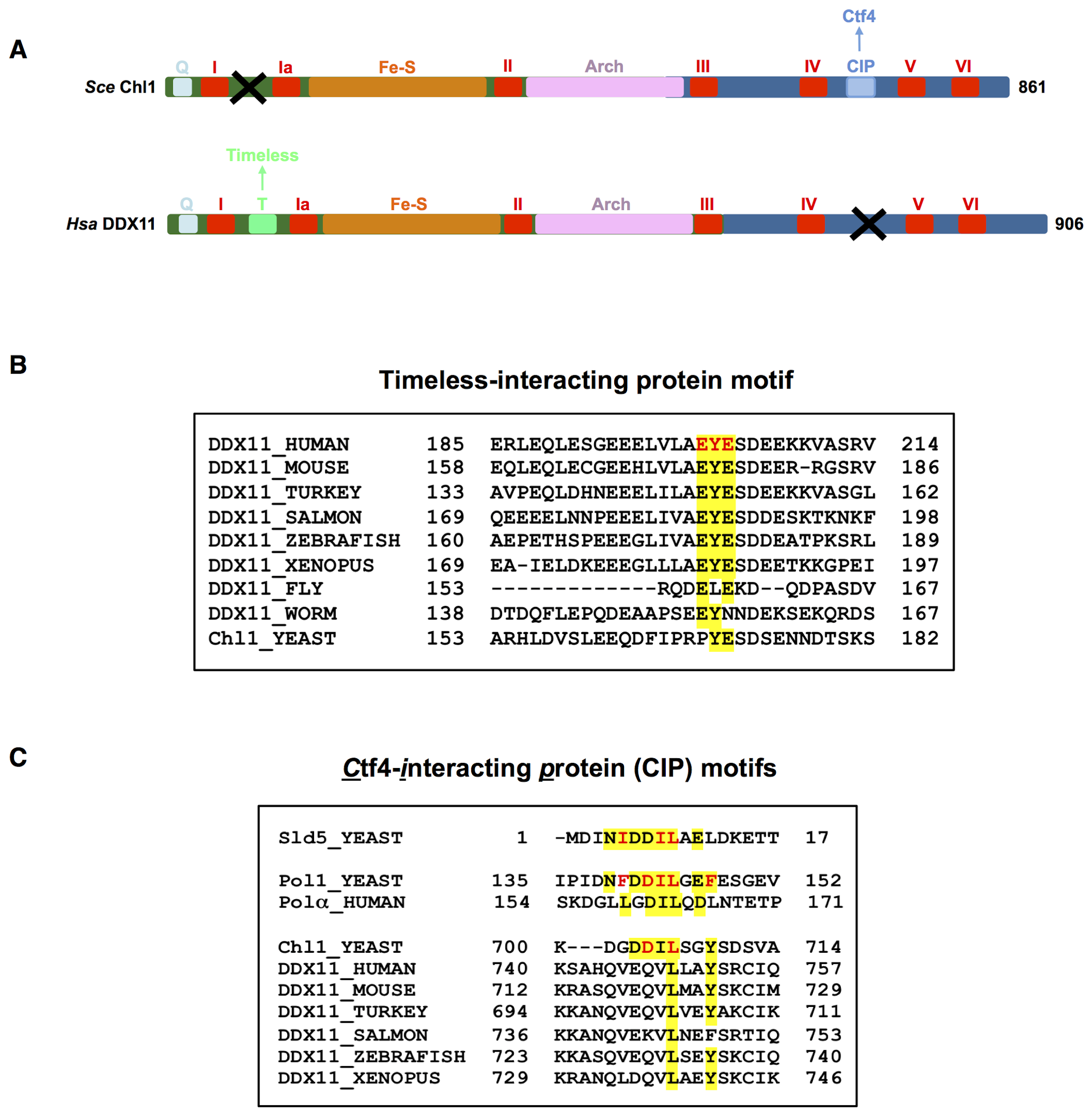

Supplement: S3 Fig — A) Schematic representation of the polypeptide chain of Homo sapiens (Hsa) DDX11 and Saccharomyces cerevisiae (Sce) Chl1, both belonging to the group of SF2 DNA helicases with a Fe-S cluster. Conserved helicase motifs (from I to VI) are indicated in red. Other sequence motifs are indicated with different colours. Abbreviations used are: Q, for Q motif; Fe-S, for Fe-S cluster; Arch, for Arch domain; CIP, for Ctf4-interacting protein motif; T, for Timeless-interacting Region T. B) Multiple alignment of the putative DDX11/Chl1 "EYE" motif from various organisms. Amino acids shown to be essential for interaction with Timeless in human DDX11 are in red. C) Alignment of putative CIP motifs of various yeast and human proteins and their partial conservation in vertebrate DDX11 orthologs. Pol1_YEAST stands for Saccharomyces cerevisiae DNA polymerase 1 catalytic subunit; Pol α_HUMAN stands for Homo sapiens DNA polymerase α p180 subunit. Amino acids shown to be essential for interaction with Ctf4 n budding yeast proteins are in red [36, 44]. Highly conserved residues in the aligned sequences are highlighted in yellow. In B and C the aligned sequences are from the following species: Homo sapiens (HUMAN), Mus musculus (MOUSE), Meleagris gallopavo (TURKEY), Salmo salar (SALMON), Danio rerio (ZEBRAFISH), Xenopus laevis (XENOPUS), Drosophila melanogaster (FLY), Caenorhabditis elegans (WORM), Saccharomyces cerevisiae (YEAST). (TIFF) [file pgen.1007622.s003.tiff]

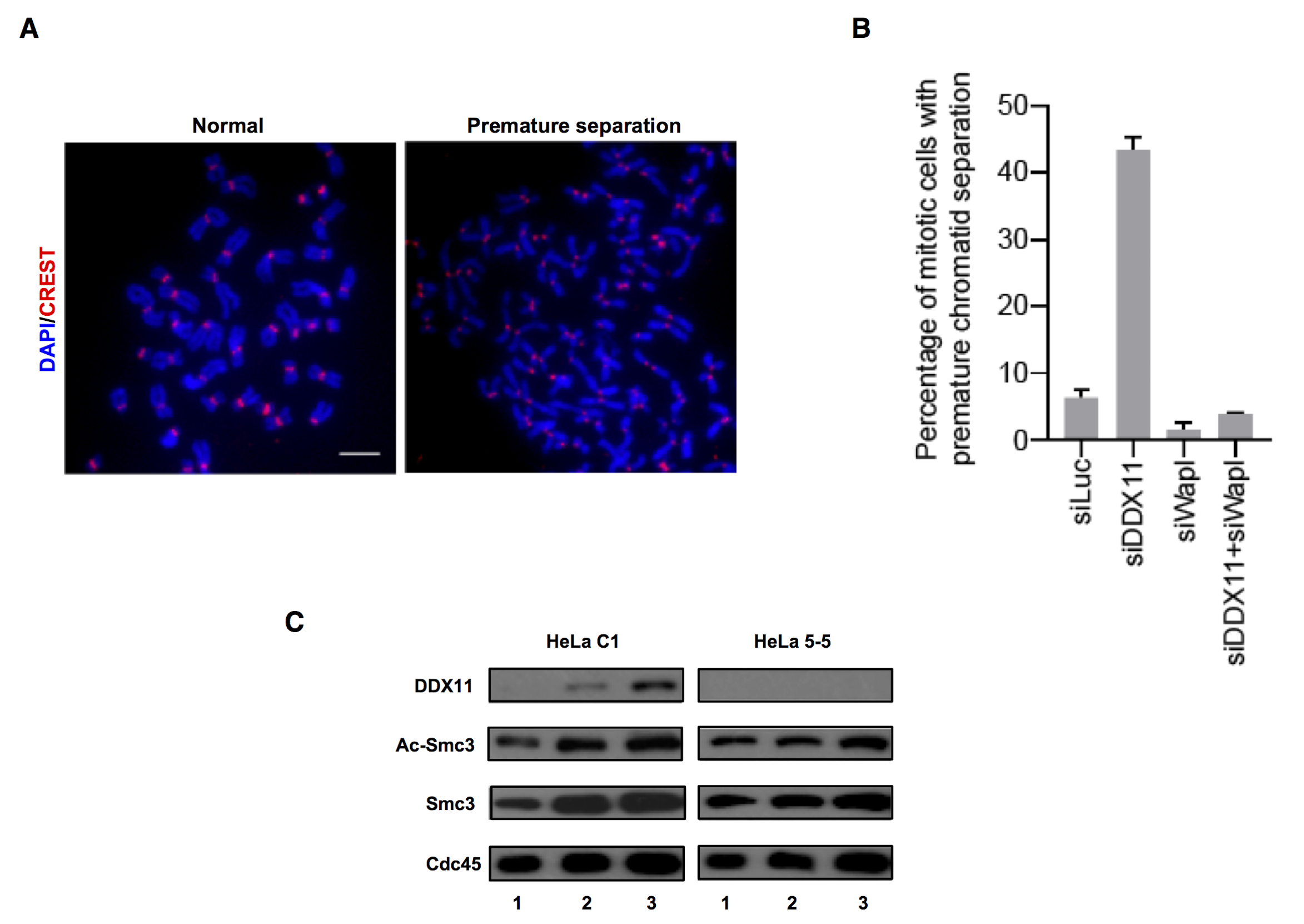

Supplement: S4 Fig — A) Analysis of metaphase chromosome spreads in DDX11/Wapl co-depleted HeLa cells. Representative images of HeLa cells with not separated or prematurely separated chromatids. Metaphase spreads were stained with DAPI (blue) and the kinetochore marker CREST (red). Scale bar, 5 μm. B) Quantification of HeLa cells with premature chromatid separation. Cells were transfected with the indicated siRNAs and enriched in mitosis. Sequence of the siRNA used to downregulate Wapl was: 5'-CGGACTACCCTTAGCACAA-3'. C) Level of acetylated Smc3 in DDX11-depleted HeLa cells. Immuno-blot showing level of the indicated proteins in HeLa cells expressing DDX11-shRNA (HeLa 5–5) and control line (HeLa C1). Samples containing 4 μg (lane 1), 6 μg (lane 2) and 8 μg (lane 3) of total protein present in the cell extract nuclear fraction were employed for Western blot analyses. Mouse monoclonal antibodies against Smc3 acetylated peptide 97-SLRRVIGAKAcKAcDQYFLDKKMC-116 and against the same not-acetylated peptide (a gift of Katsuhiko Shirahige, Tokyo, Japan) were used for detection. All samples were run on the same gel and blots were cut horizontally. Blots used to detect acetylated Smc3 were stripped and subsequently probed for Smc3. The relative amount of acetylated Smc3 (normalized to total Smc3) in HeLa C1 and 5–5 cells was quantified by comparing chemiluminescent signal intensities using the program ImageJ. Analyses were carried out in triplicate and median values of Ac-Smc3/Smc3 were: 0.93 ± 0.07 (HeLa C1) and 0.86 ± 0.05 (HeLa 5–5). (TIFF) [file pgen.1007622.s004.tiff]

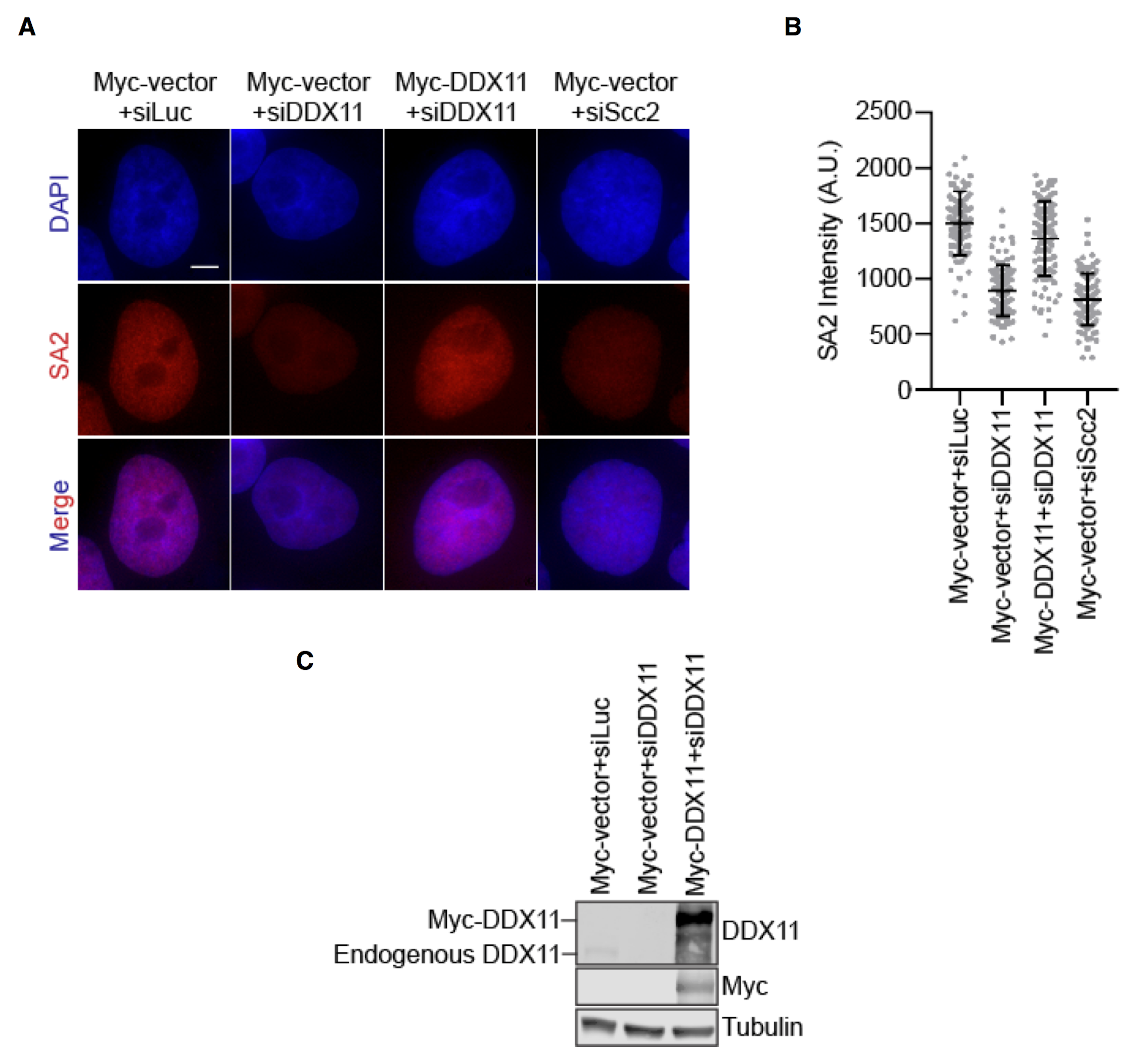

Supplement: S5 Fig — A) DAPI (blue) and anti-SA2 (red) staining of HeLa cells transfected with the indicated plasmids and siRNAs and arrested in early S phase with thymidine. Scale bar, 5 μm. B) Quantification of the SA2 chromatin intensities of cells in A. Each dot in the graph represents a single cell. Mean values and standard deviations (Myc-vector/siLuc, n = 115; Myc-vector/siDDX11, n = 102; Myc-DDX11/siDDX11, n = 97; Myc-vector/siScc2, n = 84). According to Student’s t-test, a value of P < 0.0001 was calculated for Myc-vector/siDDX11 versus Myc-DDX11/siDDX11. C) Extracts of HeLa cells transfected with the indicated plasmids and siRNAs were blotted with the indicated antibodies. (TIFF) [file pgen.1007622.s005.tiff]

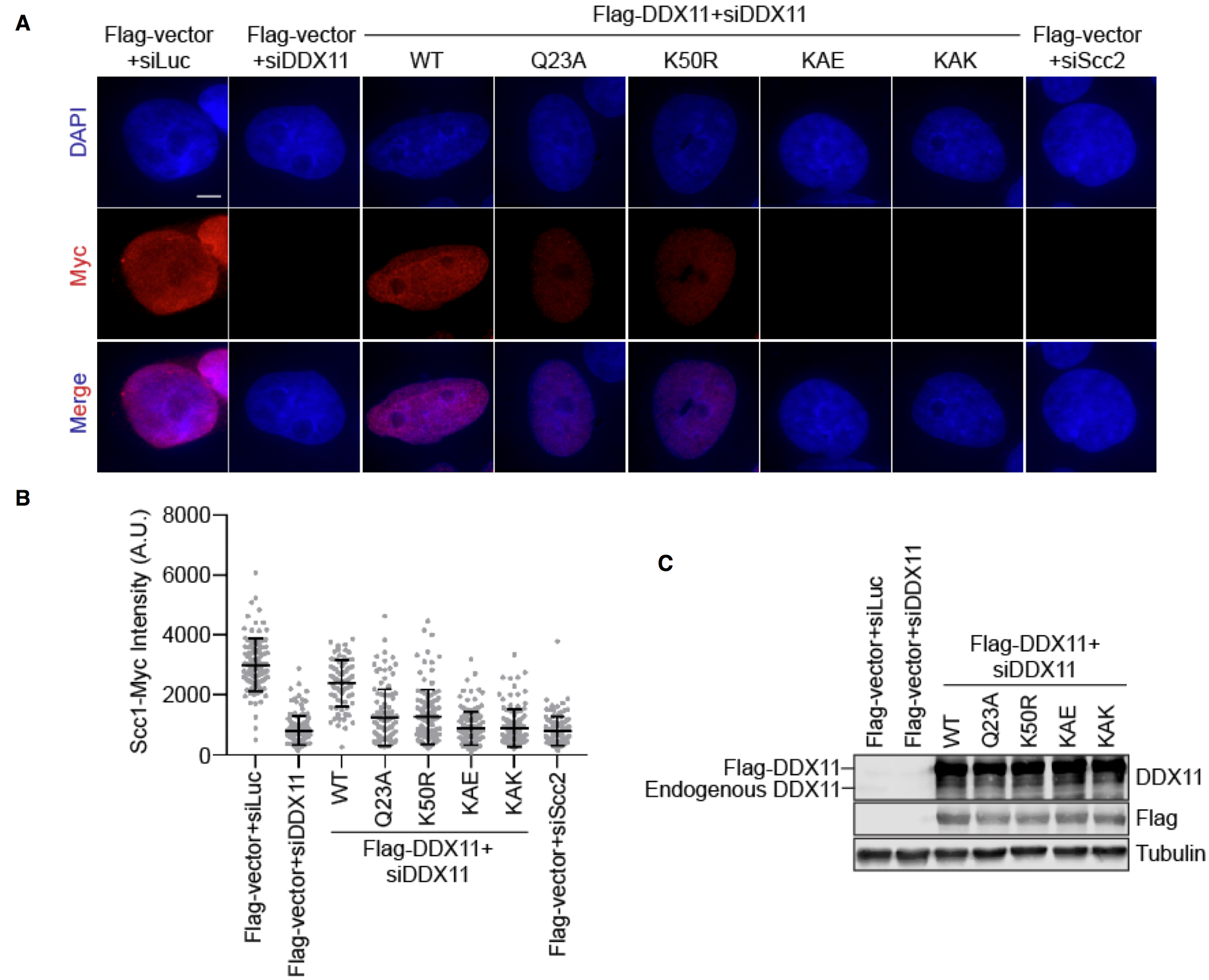

Supplement: S6 Fig — A) DAPI (blue) and anti-Myc (red) staining of HeLa cells that stably expressed Scc1-Myc. Cells were transfected with the indicated plasmids and siRNAs and arrested in early S phase with thymidine. Scale bar, 5 μm. B) Quantification of the Scc1-Myc chromatin intensities of cells in A. Each dot in the graph represents a single cell. Mean values and standard deviations (Flag-vector/siLuc, n = 103; Flag-vector/siDDX11, n = 134; WT/siDDX11, n = 81; Q23A/siDDX11, n = 102; K50R/siDDX11, n = 111; KAE/siDDX11, n = 127; KAK/siDDX11, n = 106; Flag-vector/siScc2, n = 118). According to Student’s t-test, a value of P < 0.0001 was calculated for the following dataset pairs: Flag-vector/siDDX11 versus WT/siDDX11, Q23A/siDDX11, K50R/siDDX11; WT/siDDX11 versus Q23A/siDDX11, K50R/siDDX11, KAE/siDDX11, KAK/siDDX11; K50R/siDDX11 versus KAE/siDDX11; a value of P = 0.0003 for Q23A/siDDX11 versus KAE/siDDX11; a value of P = 0.0022 for Q23A/siDDX11 versus KAK/siDDX11; a value of P = 0.0008 for K50R/siRNA versus Q23A/siDDX11. Not significant P values were calculated for the following dataset pairs: Flag vector/siDDX11 versus KAE/siDDX11 (P = 0.2722), KAK/siDDX11 (P = 0.1916); Q23A/siDDX11 versus K50R/siDDX11 (P = 0.8920); KAE/siDDX11 versus KAK/siDDX11 (P = 0.7628). C) Extracts of HeLa cells transfected with the indicated plasmids and siRNAs were probed with the indicated antibodies in Western blot experiments. (TIFF) [file pgen.1007622.s006.tiff]

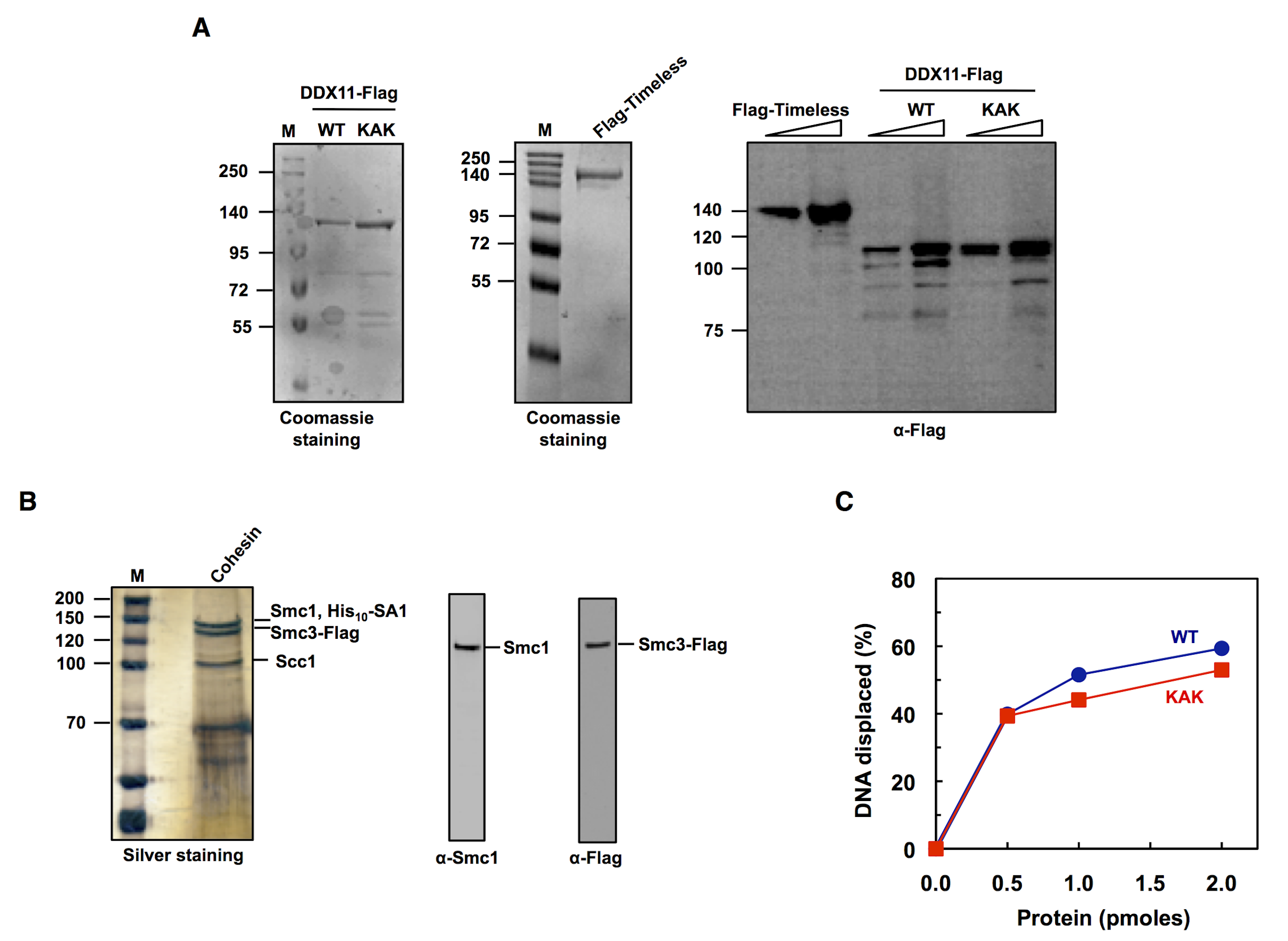

Supplement: S7 Fig — A) and B) SDS-PAGE analysis of recombinant human Timeless, purified from baculovirus-infected Sf9 insect cells; DDX11 (wild type and KAK mutant), purified from HEK 293T cells transiently transfected with pcDNA 3.0 vector derivatives; cohesin core complex, purified from baculovirus-infected Sf9 cells. Purification procedures are described in the Materials and Methods section. M indicates lane containing protein markers. Western blot analysis of purified recombinant Timeless, DDX11 WT and KAK mutant (50 and 100 ng of each protein sample) and purified cohesin complex (250 ng) were carried out using the indicated antibodies. C) Plot showing DNA helicase activity of DDX11 wild type and KAK mutant. Samples were incubated for 60 min at 37°C, as previously described [28]. A radio-labelled forked duplex DNA was used as substrate. Gels were analysed using a phosphorimaging system. (TIFF) [file pgen.1007622.s007.tiff]

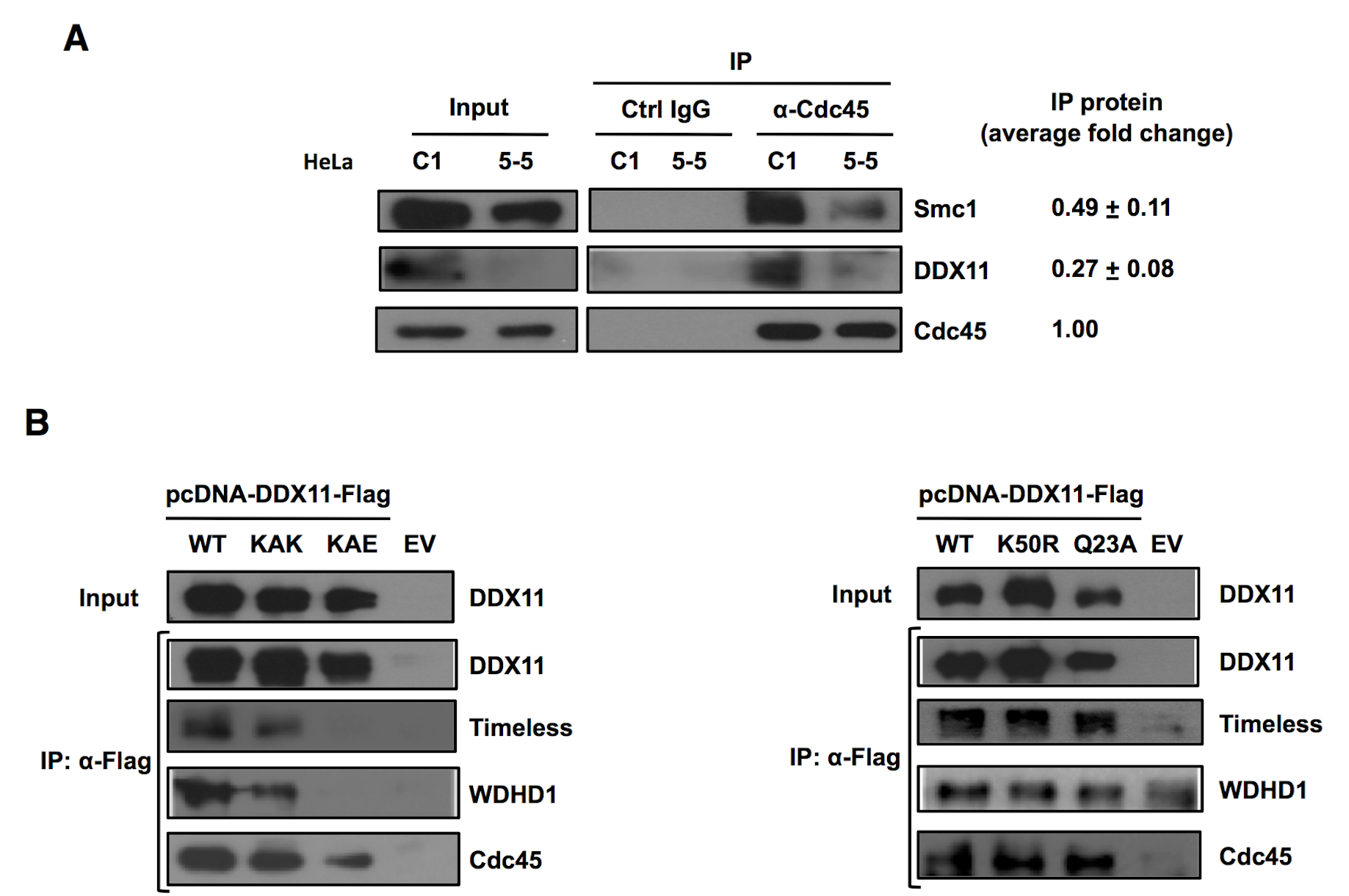

Supplement: S8 Fig — A) DDX11 and the cohesin complex are associated with the replication machinery on chromatin in S phase cells. IP experiments were carried out on the nuclear fraction of control (C1) and DDX11-downregulated (5–5) HeLa cells with control and anti-Cdc45 rabbit IgG bound to Protein A Sepharose beads. The immuno-precipitated samples were analyzed by Western blot to detect the indicated proteins. Experiments were carried out in duplicate and level of the immuno-precipitated proteins (median values with standard errors) are indicated. Reported values were normalized to the level of Cdc45 pulled down in each sample. Quantitative analyses of immuno-blot signals were carried out using the ImageJ software. B) Interaction of DDX11 with replisome components in cell extracts. IP experiments were carried out on extracts from HEK 293T cells transiently transfected with an empty vector (EV) and vectors over-expressing Flag-tagged wild type DDX11 (WT) and its mutant derivatives (KAK and KAE, on left; and K50R and Q23A, on right). Anti-Flag M2 agarose beads were used. (TIFF) [file pgen.1007622.s008.tiff]
